# Supplementary material for: Preparation of Key Intermediates for the Syntheses of Coenzyme Q10 and Derivatives by Cross-Metathesis Reactions
Source: Molecules. 2020 Jan 21;25(3):448. doi: 10.3390/molecules25030448 (PMC7036988; doi:10.3390/molecules25030448)

Supplementary Material

Preparation of key intermediates for the synthesis of Coenzyme Q_{10} and derivatives via cross-metathesis reactions

Trang Thu Thi Nguyen,[1] and Hung Dinh Mac [2,] * and Phong Van Pham[1,] *

[1] *Laboratory of Catalysis at Faculty of Chemistry, VNU University of Science, Vietnam National University, Hanoi 110403, Vietnam*

[2] *Laboratory of Pharmaceutical Chemistry at Faculty of Chemistry, VNU University of Science, Vietnam National University, Hanoi 110403, Vietnam*

Email: phvpham@hus.edu.vn

Table of Contents

IR Spectrum of 4-(allyoxy)-2,3,6-trimethylphenyl acetate (**5a**)	S4
^{1}H NMR Spectrum (500 MHz, $CDCl_3$) of 4-(allyoxy)-2,3,6-trimethylphenyl acetate (**5a**)	S4
^{13}C NMR Spectrum (125 MHz, $CDCl_3$) of 4-(allyoxy)-2,3,6-trimethylphenyl acetate (**5a**)	S5
IR Spectrum of 4-(allyloxy)-2-methylnaphthalen-1-yl acetate (**5d**)	S5
^{1}H NMR Spectrum (500 MHz, $CDCl_3$) of 4-(allyloxy)-2-methylnaphthalen-1-yl acetate (**5d**)	S6
^{13}C NMR Spectrum (125 MHz, $CDCl_3$) of 4-(allyloxy)-2-methylnaphthalen-1-yl acetate (**5d**)	S6
IR Spectrum of 3-allyl-4-hydroxy-2,5,6-trimethylphenyl acetate (**6a**)	S7
^{1}H NMR Spectrum (500 MHz, $CDCl_3$) of 3-allyl-4-hydroxy-2,5,6-trimethylphenyl acetate (**6a**)	S7
^{13}C NMR Spectrum (125 MHz, $CDCl_3$) of 3-allyl-4-hydroxy-2,5,6-trimethylphenyl acetate (**6a**)	S8
IR Spectrum of 3-allyl-4-hydroxy-2-methylnaphthalen-1-yl acetate (**6d**)	S8
^{1}H NMR Spectrum (500 MHz, $CDCl_3$) of 3-allyl-4-hydroxy-2-methylnaphthalen-1-yl acetate (**6d**)	S9
^{13}C NMR Spectrum (125 MHz, $CDCl_3$) of 3-allyl-4-hydroxy-2-methylnaphthalen-1-yl acetate (**6d**)	S9
IR Spectrum of 2-allyl-3,5,6-trimethyl-1,4-phenylene diacetate (**7a**)	S10
^{1}H NMR Spectrum (500 MHz, $CDCl_3$) of 2-allyl-3,5,6-trimethyl-1,4-phenylene diacetate (**7a**)	S10
^{13}C NMR Spectrum (125 MHz, $CDCl_3$) of 2-allyl-3,5,6-trimethyl-1,4-phenylene diacetate (**7a**)	S11
IR Spectrum of 1-allyl-2,5-dimethoxy-3,4,6-trimethylbenzene (**7b**)	S11
^{1}H NMR Spectrum (500 MHz, $CDCl_3$) of 1-allyl-2,5-dimethoxy-3,4,6-trimethylbenzene (**7b**)	S12
^{13}C NMR Spectrum (125 MHz, $CDCl_3$) of 1-allyl-2,5-dimethoxy-3,4,6-trimethylbenzene (**7b**)	S12
IR Spectrum of 1-allyl-2,3,4,5-tetramethoxy-6-methylbenzene (**7c**)	S13
^{1}H NMR Spectrum (500 MHz, $CDCl_3$) of 1-allyl-2,3,4,5-tetramethoxy-6-methylbenzene (**7c**)	S13
^{13}C NMR Spectrum (125 MHz, $CDCl_3$) of 1-allyl-2,3,4,5-tetramethoxy-6-methylbenzene (**7c**)	S14
IR Spectrum (500 MHz, $CDCl_3$) of 2-allyl-3-methylnaphthalene-1,4-diyl diacetate (**7d**)	S14
^{1}H NMR Spectrum (500 MHz, $CDCl_3$) of 2-allyl-3-methylnaphthalene-1,4-diyl diacetate (**7d**)	S15

^{13}C NMR Spectrum (125 MHz, $CDCl_3$) of 2-allyl-3-methylnaphthalene-1,4-diyl diacetate (**7d**)	S15
^{1}H NMR Spectrum (500 MHz, $CDCl_3$) of 2-allyl-1,4-dimethoxy-3-methylnaphthalene (**7e**)	S16
^{13}C NMR Spectrum (125 MHz, $CDCl_3$) of 2-allyl-1,4-dimethoxy-3-methylnaphthalene (**7e**)	S16
IR Spectrum of (*E*)-2-(4-methoxy-3-methyl-4-oxobut-2-en-1-yl)-3,5,6-trimethyl-1,4-phenylene diacetate (**8a**)	S17
^{1}H NMR Spectrum (500 MHz, $CDCl_3$) of (*E*)-2-(4-methoxy-3-methyl-4-oxobut-2-en-1-yl)-3,5,6-trimethyl-1,4-phenylene diacetate (**8a**)	S17
^{13}C NMR Spectrum (125 MHz, $CDCl_3$) of (*E*)-2-(4-methoxy-3-methyl-4-oxobut-2-en-1-yl)-3,5,6-trimethyl-1,4-phenylene diacetate (**8a**)	S18
Noesy spectrum of (*E*)-2-(4-methoxy-3-methyl-4-oxobut-2-en-1-yl)-3,5,6-trimethyl-1,4-phenylene diacetate (**8a**)	S18
IR Spectrum of methyl (*E*)-4-(2,5-dimethoxy-3,4,6-trimethylphenyl)-2-methylbut-2-enoate (**8b**)	S19
^{1}H NMR Spectrum (500 MHz, $CDCl_3$) of methyl (*E*)-4-(2,5-dimethoxy-3,4,6-trimethylphenyl)-2-methylbut-2-enoate (**8b**)	S19
^{13}C NMR Spectrum (125 MHz, $CDCl_3$) of methyl (*E*)-4-(2,5-dimethoxy-3,4,6-trimethylphenyl)-2-methylbut-2-enoate (**8b**)	S20
NOESY spectrum of methyl (*E*)-4-(2,5-dimethoxy-3,4,6-trimethylphenyl)-2-methylbut-2-enoate (**8b**)	S20
IR Spectrum of methyl (*E*)-2-methyl-4-(2,3,4,5-tetramethoxy-6-methylphenyl)but-2-enoate (**8c**)	S21
^{1}H NMR Spectrum (500 MHz, $CDCl_3$) of methyl (*E*)-2-methyl-4-(2,3,4,5-tetramethoxy-6-methylphenyl)but-2-enoate (**8c**)	S21
^{13}C NMR Spectrum (125 MHz, $CDCl_3$) of methyl (*E*)-2-methyl-4-(2,3,4,5-tetramethoxy-6-methylphenyl)but-2-enoate (**8c**)	S22
IR Spectrum of (*E*)-2-(4-methoxy-3-methyl-4-oxobut-2-en-1-yl)-3-methylnaphthalene-1,4-diyl diacetate (**8d**)	S22
^{1}H NMR Spectrum (500 MHz, $CDCl_3$) of (*E*)-2-(4-methoxy-3-methyl-4-oxobut-2-en-1-yl)-3-methylnaphthalene-1,4-diyl diacetate (**8d**)	S23
^{13}C NMR Spectrum (125 MHz, $CDCl_3$) of (*E*)-2-(4-methoxy-3-methyl-4-oxobut-2-en-1-yl)-3-methylnaphthalene-1,4-diyl diacetate (**8d**)	S23
IR Spectrum of methyl (*E*)-4-(1,4-dimethoxy-3-methylnaphthalen-2-yl)-2-methylbut-2-enoate **(8e)**	S24
^{1}H NMR Spectrum (500 MHz, $CDCl_3$) of methyl (*E*)-4-(1,4-dimethoxy-3-methylnaphthalen-2-yl)-2-methylbut-2-enoate (**8e**)	S24
^{13}C NMR Spectrum (125 MHz, $CDCl_3$) of methyl (*E*)-4-(1,4-dimethoxy-3-methylnaphthalen-2-yl)-2-methylbut-2-enoate (**8e**)	S25

IR Spectrum of (*E*)-4-(2,5-dimethoxy-3,4,6-trimethylcyclohexa-1,5-dienyl)-2-methylbut-2-en-1-ol (**2b**)	S25
^{1}H NMR Spectrum (500 MHz, $CDCl_3$) of (*E*)-4-(2,5-dimethoxy-3,4,6-trimethylcyclohexa-1,5-dienyl)-2-methylbut-2-en-1-ol (**2b**)	S26
^{13}C NMR Spectrum (125 MHz, $CDCl_3$) of (*E*)-4-(2,5-dimethoxy-3,4,6-trimethylcyclohexa-1,5-dienyl)-2-methylbut-2-en-1-ol (**2b**)	S26
IR Spectrum of (*E*)-2-methyl-4-(2,3,4,5-tetramethoxy-6-methylphenyl)but-2-en-1-ol (**2c**)	S27
^{1}H NMR Spectrum (500 MHz, $CDCl_3$) of methyl (*E*)-2-methyl-4-(2,3,4,5-tetramethoxy-6-methylphenyl)but-2-en-1-ol (**2c**)	S27
^{13}C NMR Spectrum (125 MHz, $CDCl_3$) of (*E*)-2-methyl-4-(2,3,4,5-tetramethoxy-6-methylphenyl)but-2-en-1-ol (**2c**)	S28
IR Spectrum of (*E*)-4-(1,4-dimethoxy-3-methylnaphthalen-2-yl)-2-methylbut-2-en-1-ol (**2e**)	S28
^{1}H NMR Spectrum (500 MHz, $CDCl_3$) of (*E*)-4-(1,4-dimethoxy-3-methylnaphthalen-2-yl)-2-methylbut-2-en-1-ol (**2e**)	S29
^{13}C NMR Spectrum (125 MHz, $CDCl_3$) of (*E*)-4-(1,4-dimethoxy-3-methylnaphthalen-2-yl)-2-methylbut-2-en-1-ol (**2e**)	S29
IR Spectrum of (*E*)-1,4-dimethoxy-2,3,5-trimethyl-6-(3-methyl-4-(phenylsulfonyl)but-2-enyl)benzene (**10**)	S30
^{1}H NMR Spectrum (500 MHz, $CDCl_3$) of (*E*)-1,4-dimethoxy-2,3,5-trimethyl-6-(3-methyl-4-(phenylsulfonyl)but-2-enyl)benzene (**10**)	S30
^{13}C NMR Spectrum (125 MHz, $CDCl_3$) of (*E*)-1,4-dimethoxy-2,3,5-trimethyl-6-(3-methyl-4-(phenylsulfonyl)but-2-enyl)benzene (**10**)	S31
IR Spectrum of 1-((*2E,6E,10E,14E,18E,22E,26E,30E,34E*)-3,7,11,15,19,23,27,31,35,39-decamethyl-4-(phenylsulfonyl)tetraconta-2,6,10,14,18,22,26,30,34,38-decaenyl)-2,5-dimethoxy-3,4,6-trimethylbenzene (**11**)	S31
^{1}H NMR Spectrum (500 MHz, $CDCl_3$) of 1-((*2E,6E,10E,14E,18E,22E,26E,30E,34E*)-3,7,11,15,19,23,27,31,35,39-decamethyl-4-(phenylsulfonyl)tetraconta-2,6,10,14,18,22,26,30,34,38-decaenyl)-2,5-dimethoxy-3,4,6-trimethylbenzene (**11**)	S32
^{13}C NMR Spectrum (125 MHz, $CDCl_3$) of 1-((*2E,6E,10E,14E,18E,22E,26E,30E,34E*)-3,7,11,15,19,23,27,31,35,39-decamethyl-4-(phenylsulfonyl)tetraconta-2,6,10,14,18,22,26,30,34,38-decaenyl)-2,5-dimethoxy-3,4,6-trimethylbenzene (**11**)	S32
IR Spectrum of 1-((*2E,6E,10E,14E,18E,22E,26E,30E,34E*)-3,7,11,15,19,23,27,31,35,39-decamethyltetraconta-2,6,10,14,18,22,26,30,34,38-decaenyl)-2,5-dimethoxy-3,4,6-trimethylbenzene (**12**)	S33
^{1}H NMR Spectrum (500 MHz, $CDCl_3$) of 1-((*2E,6E,10E,14E,18E,22E,26E,30E,34E*)-3,7,11,15,19,23,27,31,35,39-decamethyltetraconta-2,6,10,14,18,22,26,30,34,38-decaenyl)-2,5-dimethoxy-3,4,6-trimethylbenzene (**12**)	S33
^{13}C NMR Spectrum (125 MHz, $CDCl_3$) of 1-((*2E,6E,10E,14E,18E,22E,26E,30E,34E*)-3,7,11,15,19,23,27,31,35,39-decamethyltetraconta-2,6,10,14,18,22,26,30,34,38-decaenyl)-2,5-dimethoxy-3,4,6-trimethylbenzene (**12**)	S34

IR Spectrum of 4-(allyoxy)-2,3,6-trimethylphenyl acetate (**5a**)

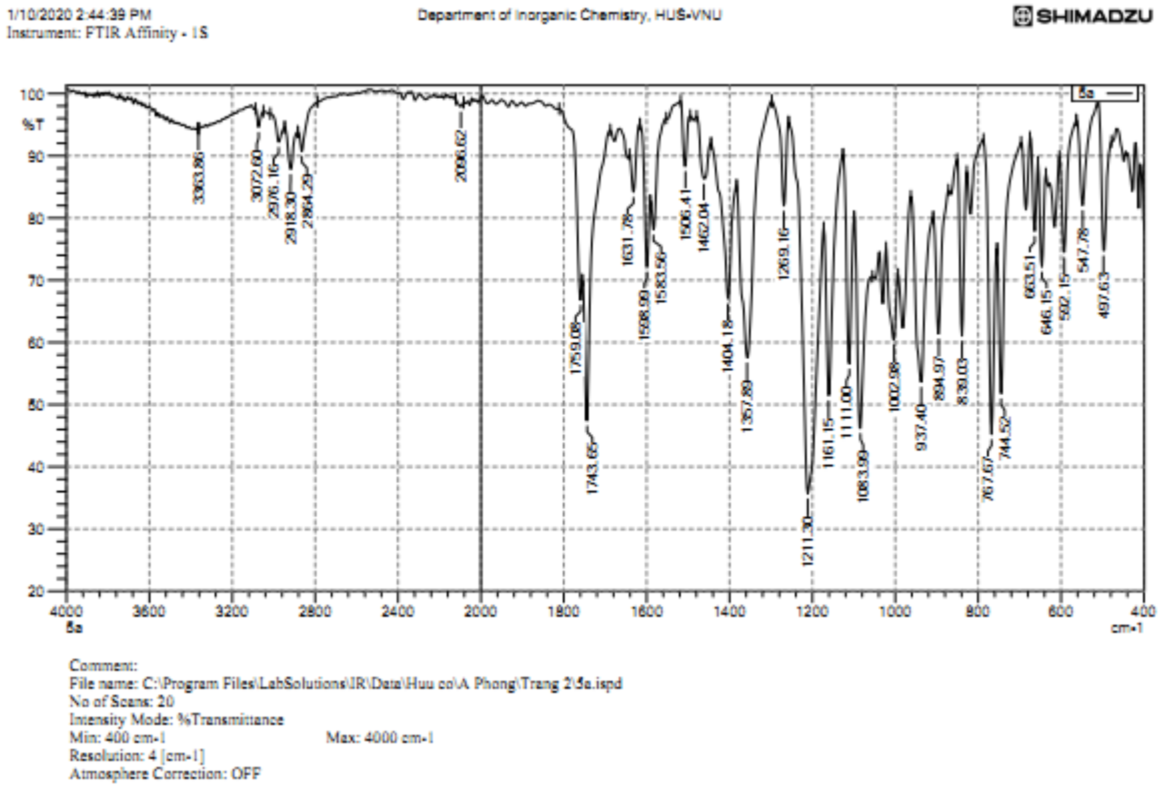

^{1}H NMR Spectrum (500 MHz, $CDCl_3$) of 4-(allyoxy)-2,3,6-trimethylphenyl acetate (**5a**)

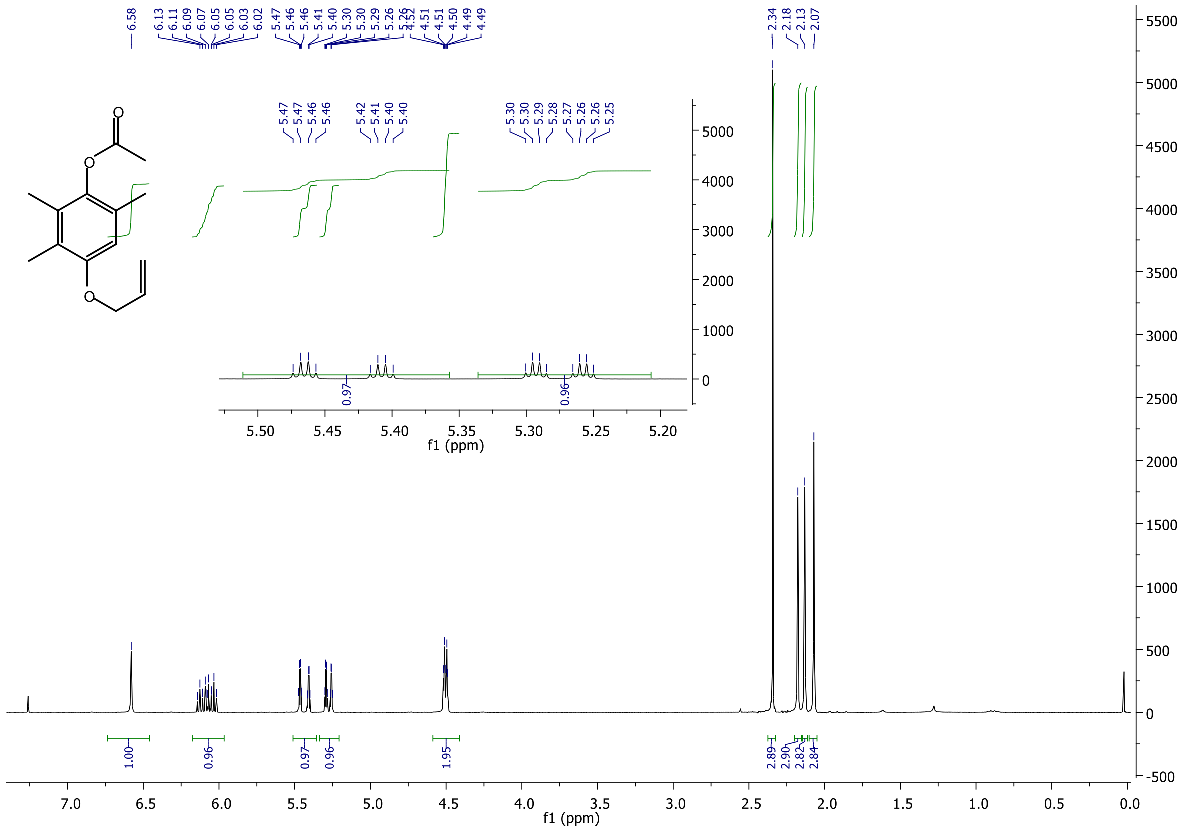

^{13}C NMR Spectrum (125 MHz, $CDCl_3$) of 4-(allyoxy)-2,3,6-trimethylphenyl acetate (**5a**)

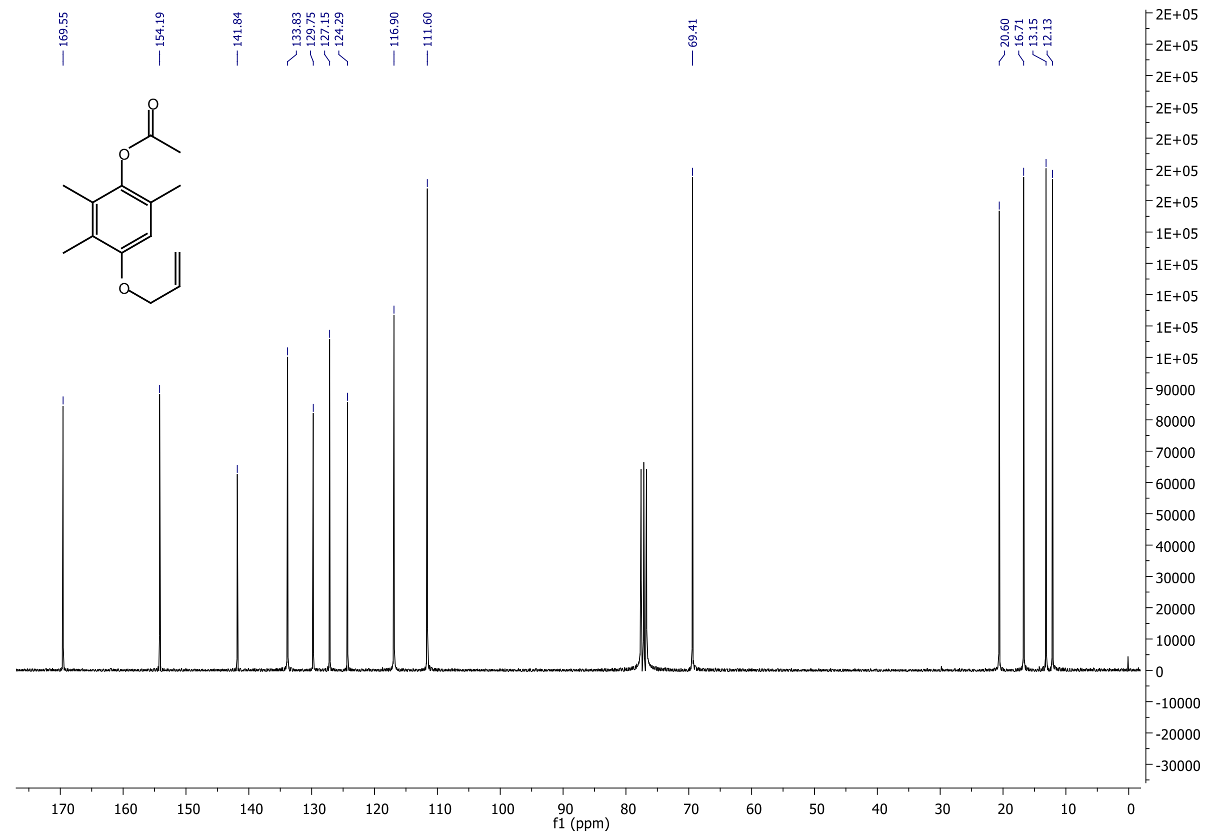

IR Spectrum of 4-(allyloxy)-2-methylnaphthalen-1-yl acetate (**5d**)

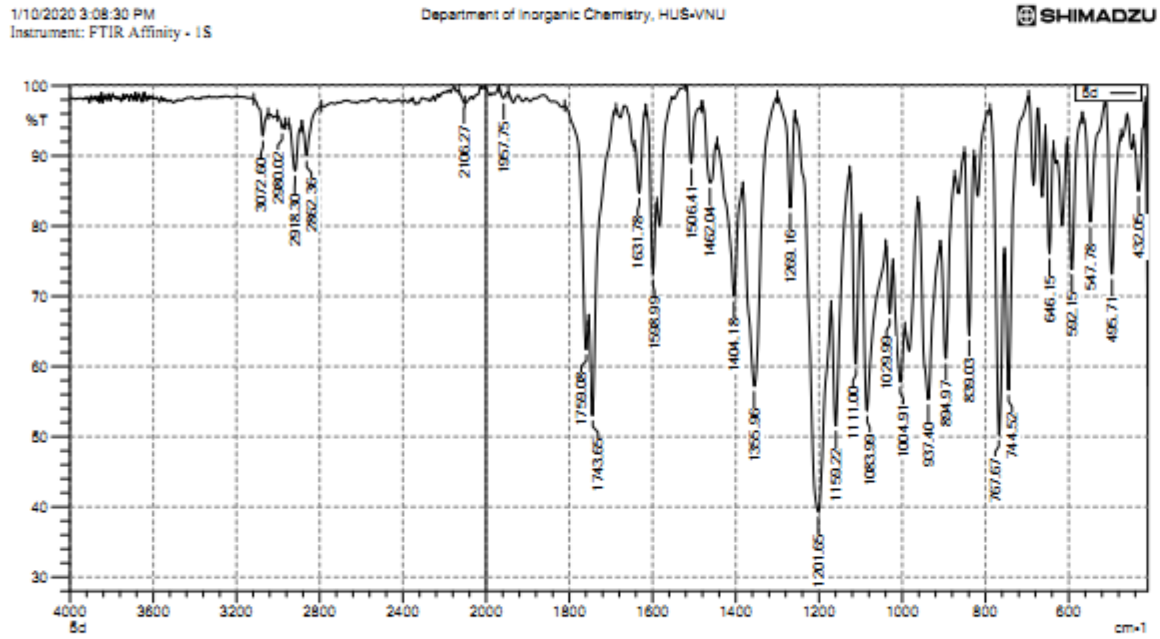

Comment:
File name: C:\Program Files\LabSolutions\IR\Data\Huu co\A Phong\Trang 2\5d.ispd
No of Scans: 20
Intensity Mode: %Transmittance
Min: 400 cm-1 Max: 4000 cm-1
Resolution: 4 [cm-1]
Atmosphere Correction: OFF

^{1}H NMR Spectrum (500 MHz, $CDCl_3$) of 4-(allyloxy)-2-methylnaphthalen-1-yl acetate (**5d**)

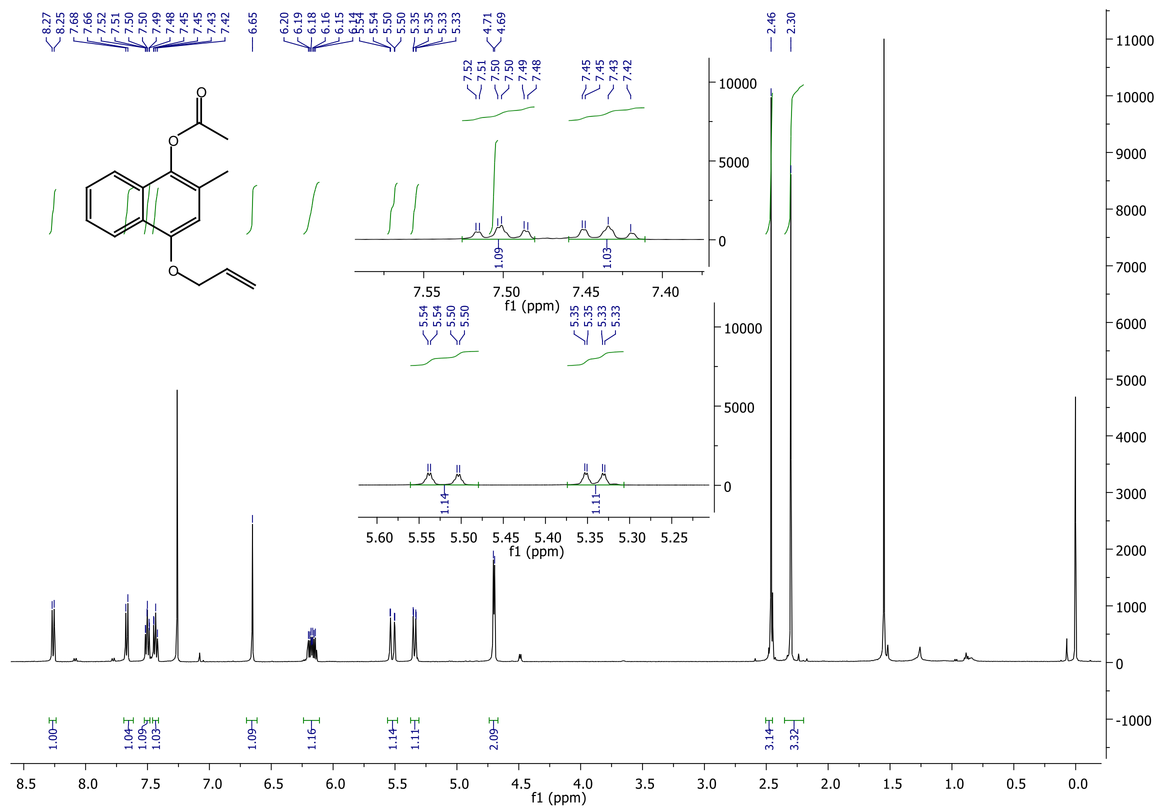

^{13}C NMR Spectrum (125 MHz, $CDCl_3$) of 4-(allyloxy)-2-methylnaphthalen-1-yl acetate (**5d**)

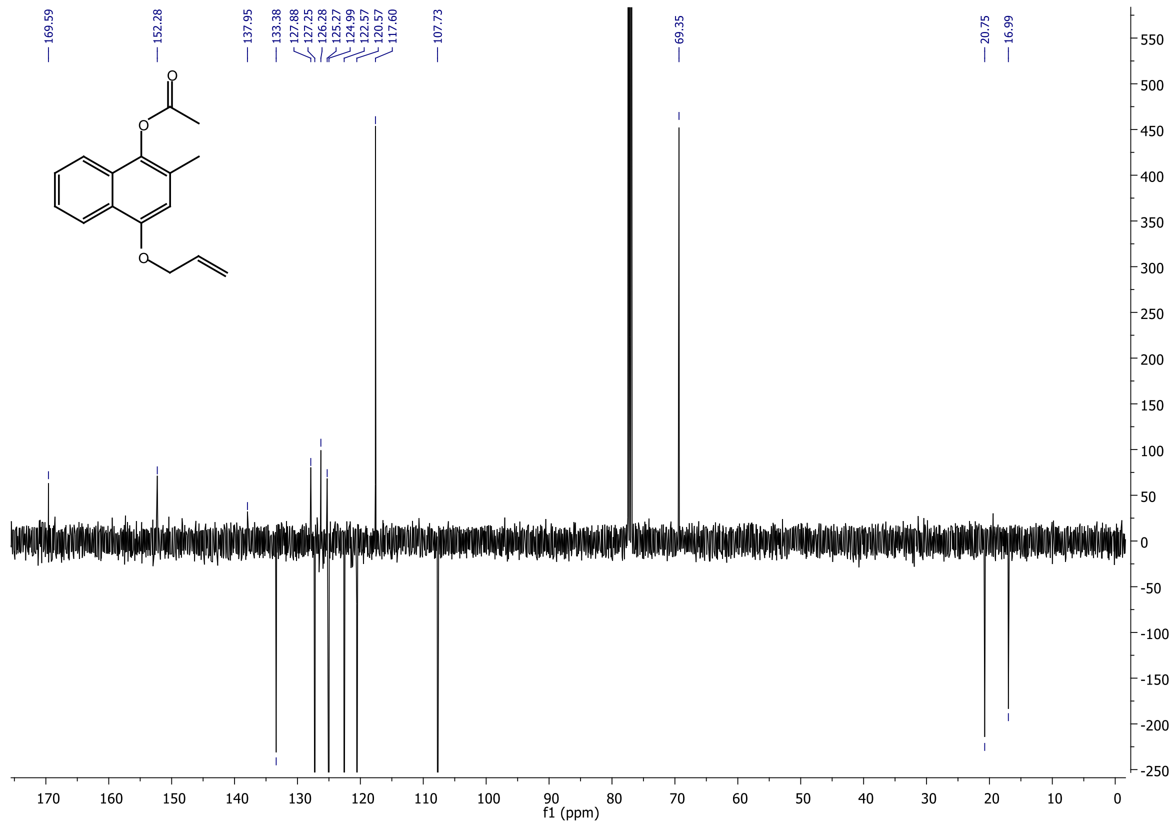

IR Spectrum of 3-allyl-4-hydroxy-2,5,6-trimethylphenyl acetate (**6a**)

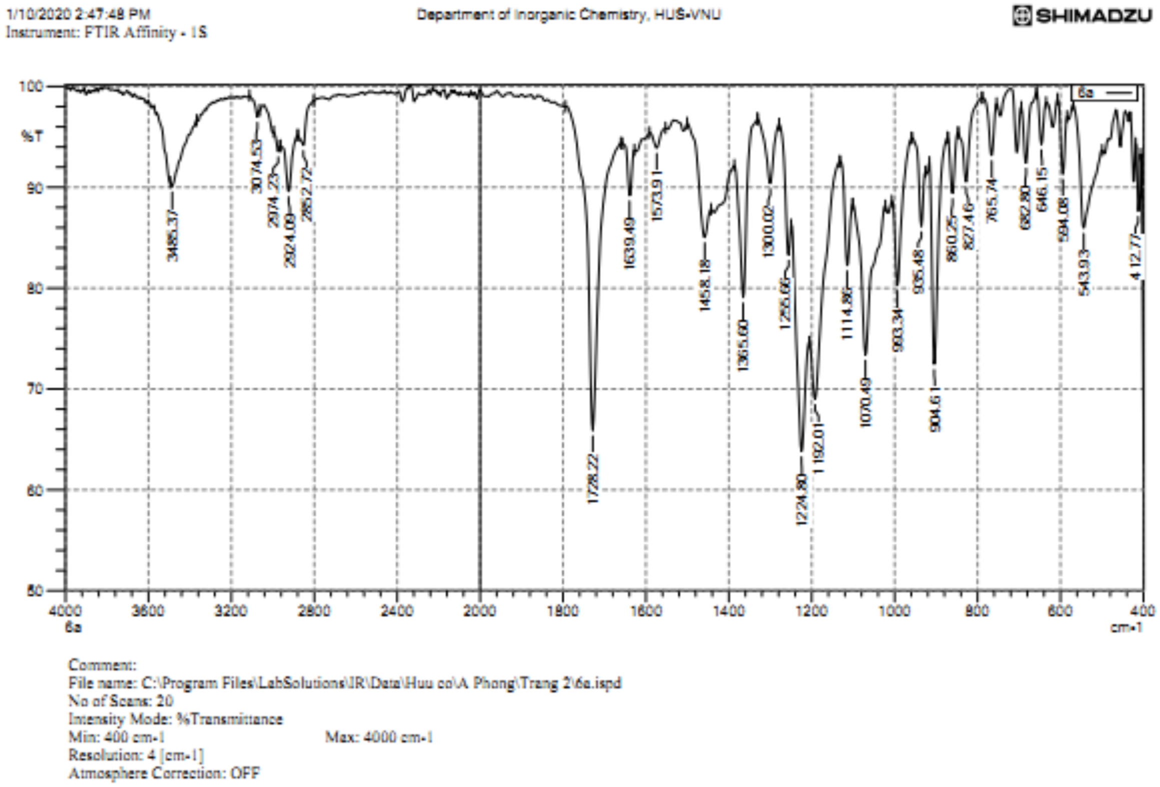

^{1}H NMR Spectrum (500 MHz, $CDCl_3$) of 3-allyl-4-hydroxy-2,5,6-trimethylphenyl acetate (**6a**)

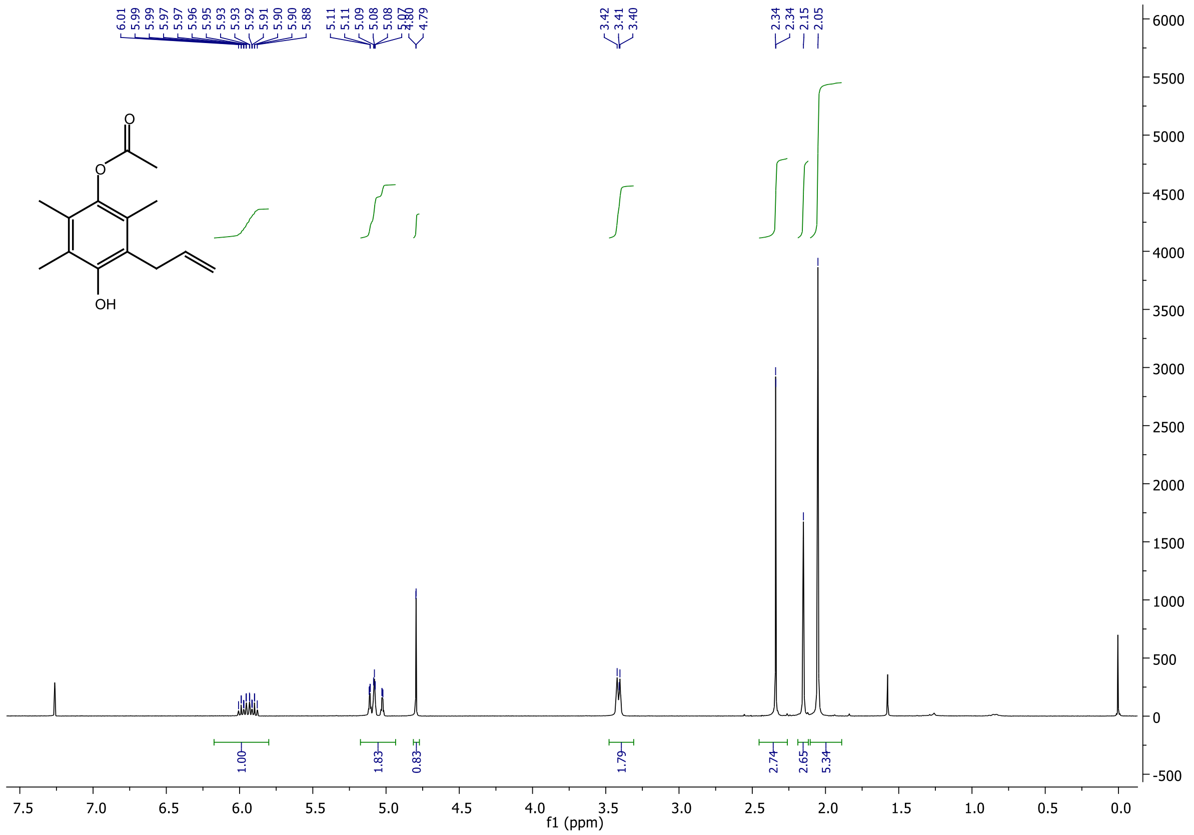

^{13}C NMR Spectrum (125 MHz, $CDCl_3$) of 3-allyl-4-hydroxy-2,5,6-trimethylphenyl acetate (**6a**)

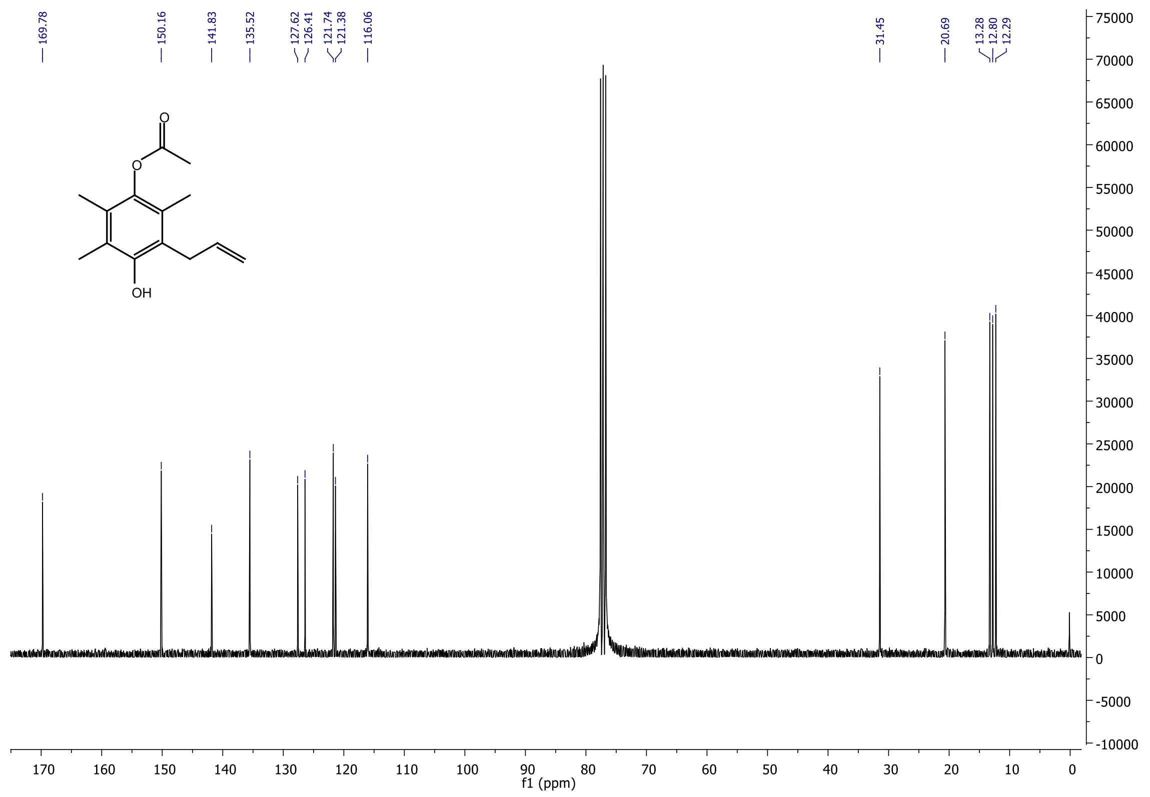

IR Spectrum of 3-allyl-4-hydroxy-2-methylnaphthalen-1-yl acetate (**6d**)

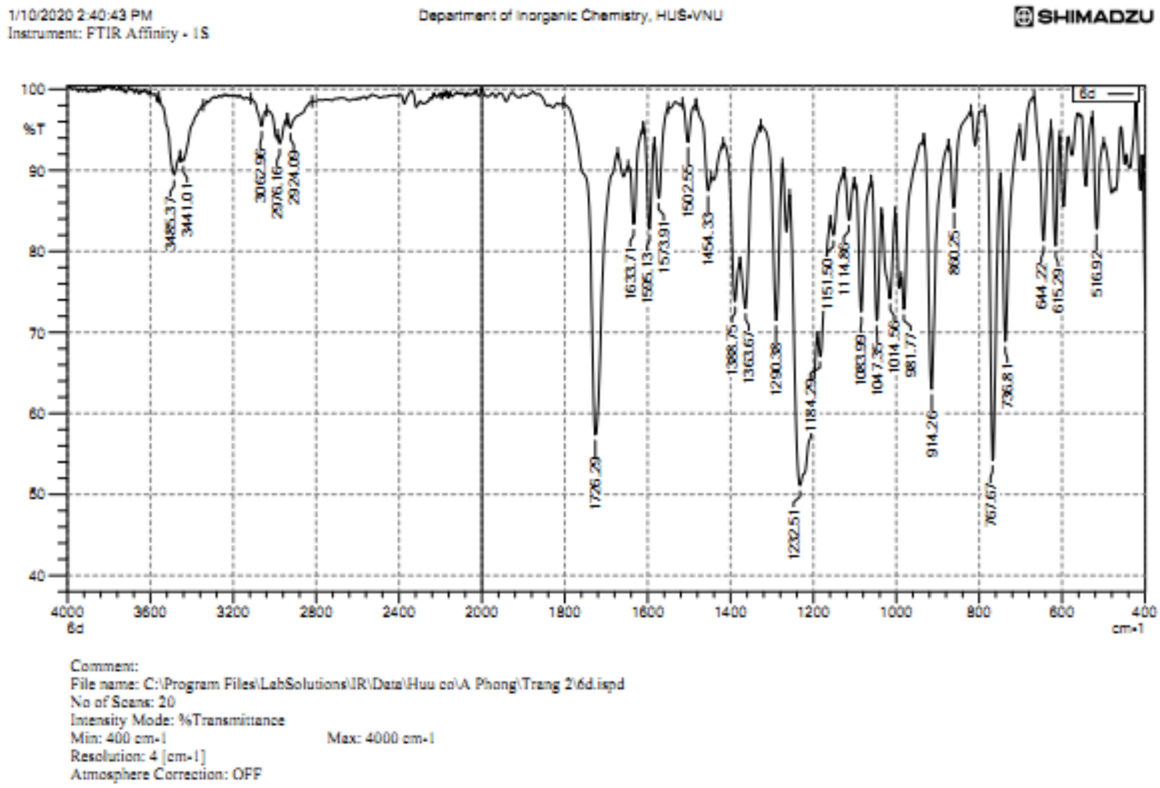

^{1}H NMR Spectrum (500 MHz, $CDCl_3$) of 3-allyl-4-hydroxy-2-methylnaphthalen-1-yl acetate (**6d**)

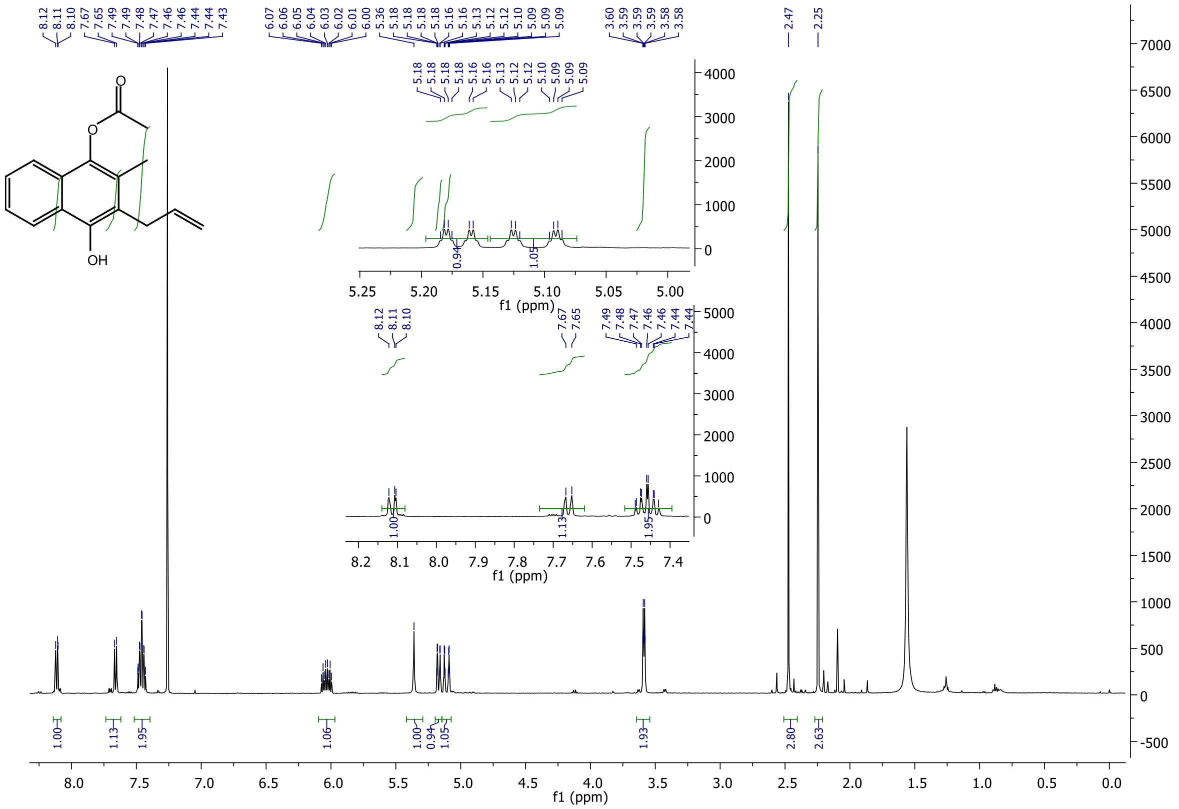

^{13}C NMR Spectrum (125 MHz, $CDCl_3$) of 3-allyl-4-hydroxy-2-methylnaphthalen-1-yl acetate (**6d**)

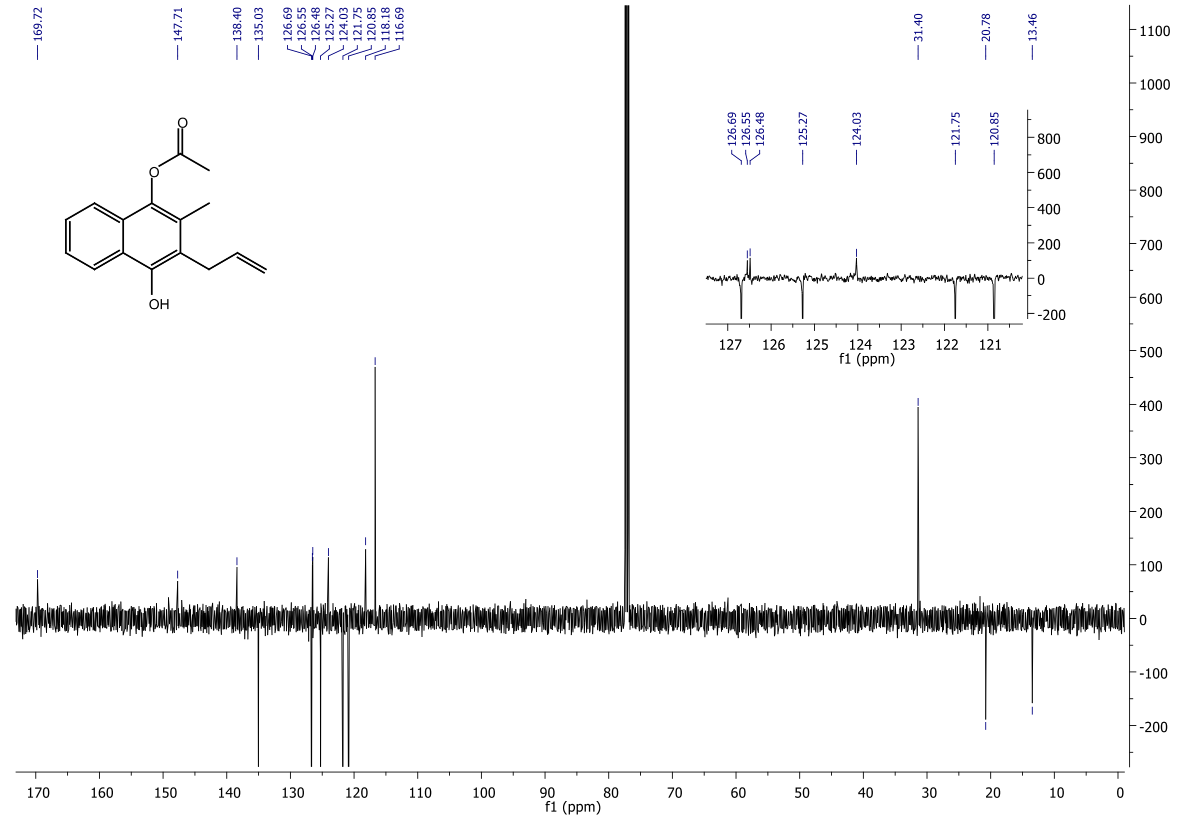

IR Spectrum of 2-allyl-3,5,6-trimethyl-1,4-phenylene diacetate (**7a**)

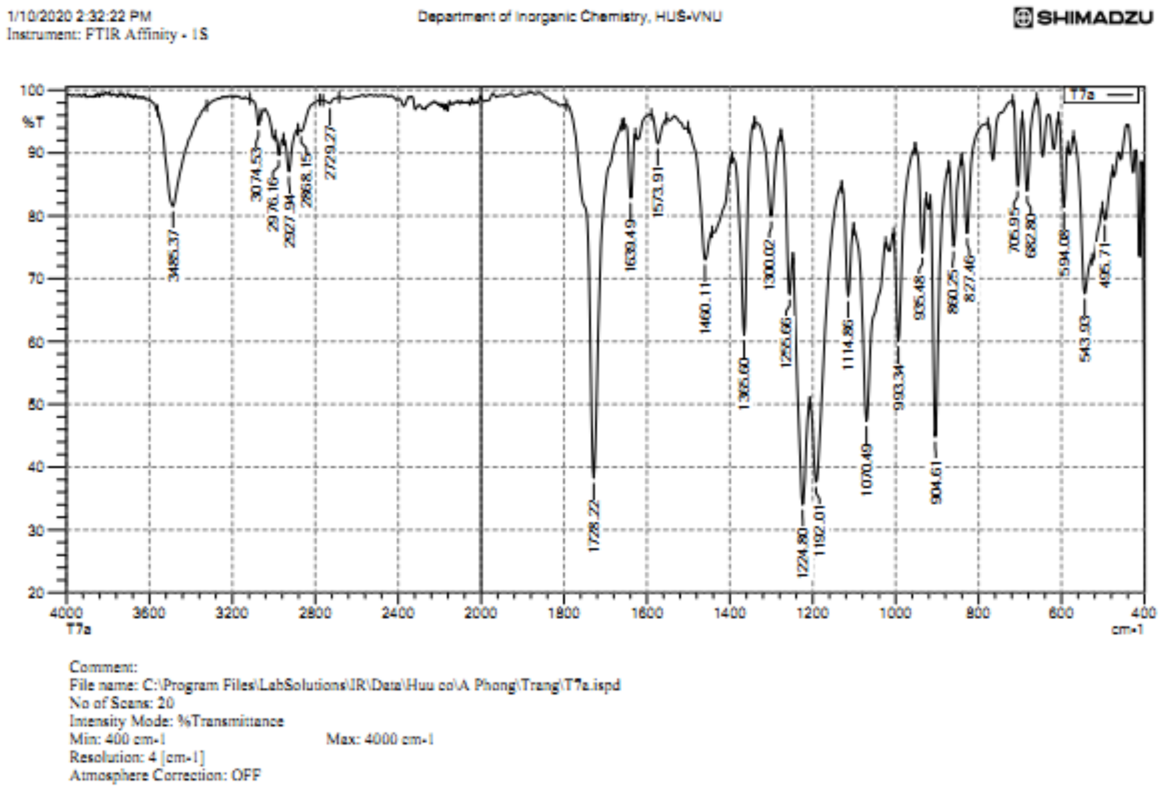

^{1}H NMR Spectrum (500 MHz, $CDCl_3$) of 2-allyl-3,5,6-trimethyl-1,4-phenylene diacetate (**7a**)

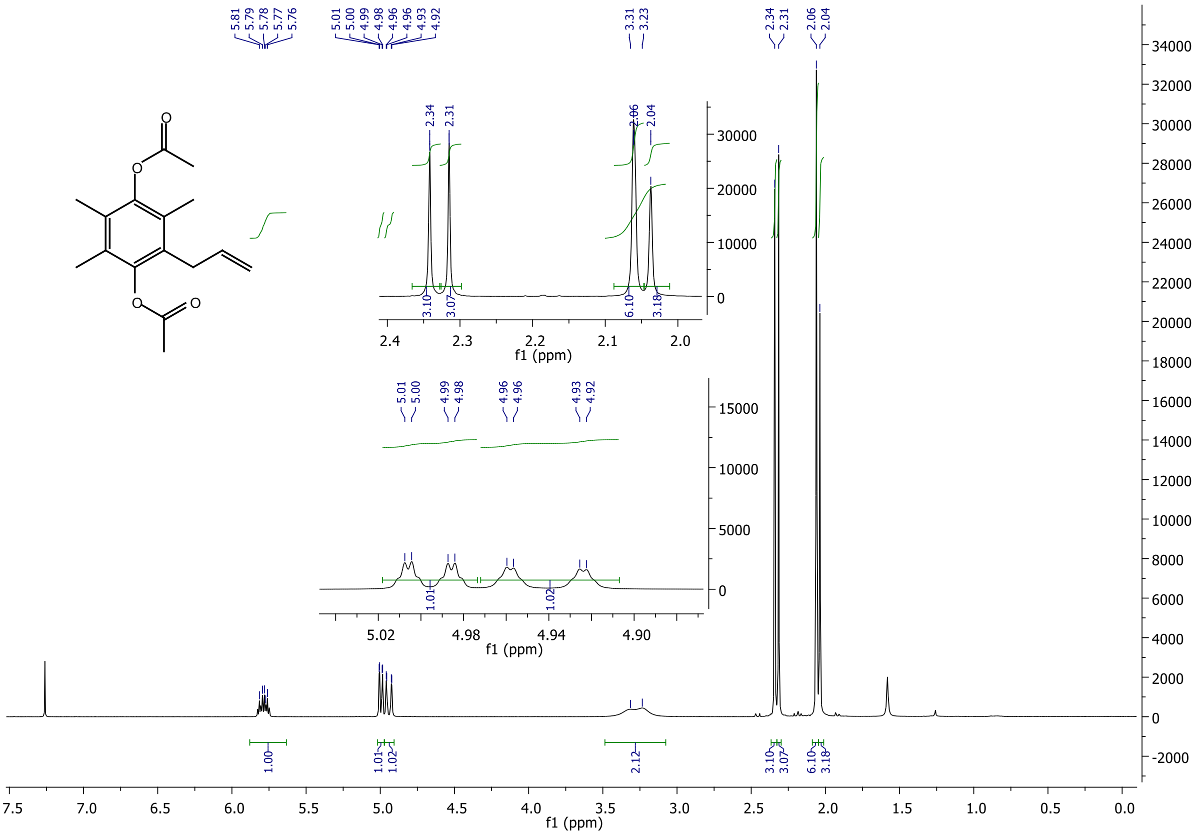

^{13}C NMR Spectrum (125 MHz, $CDCl_3$) of 2-allyl-3,5,6-trimethyl-1,4-phenylene diacetate (**7a**)

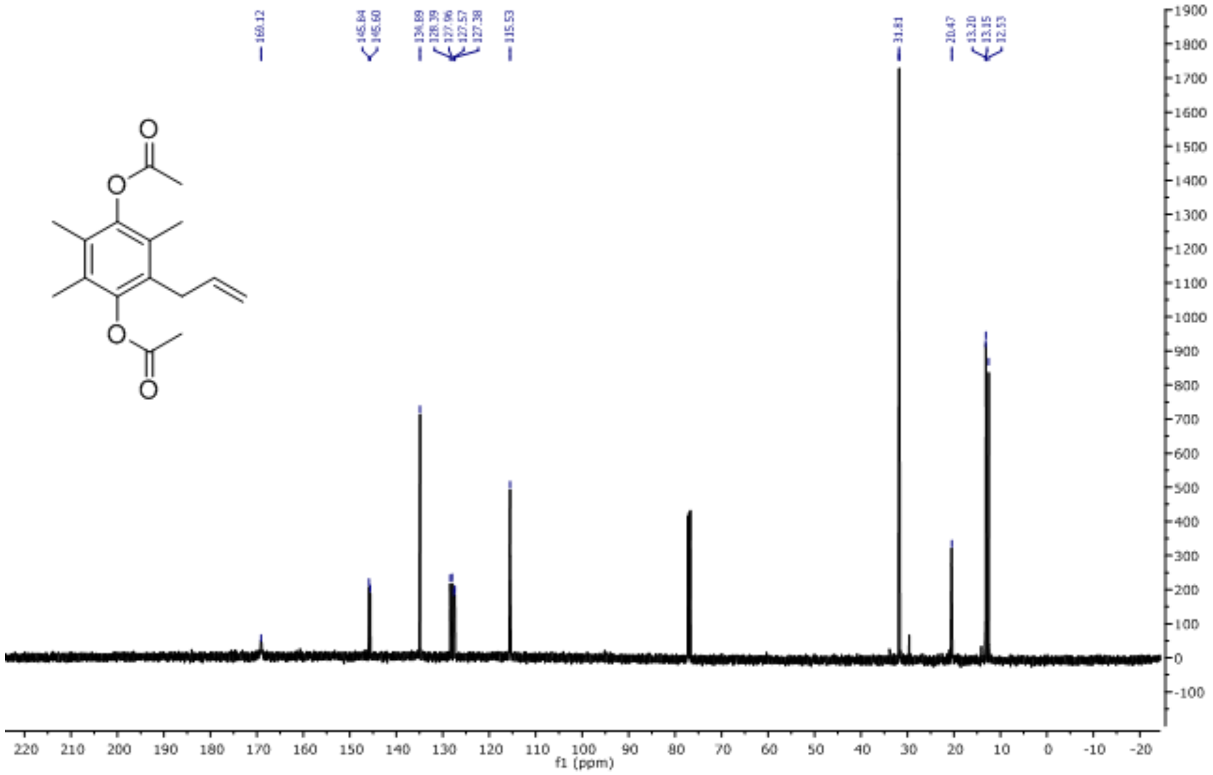

IR Spectrum of 1-allyl-2,5-dimethoxy-3,4,6-trimethylbenzene (**7b**)

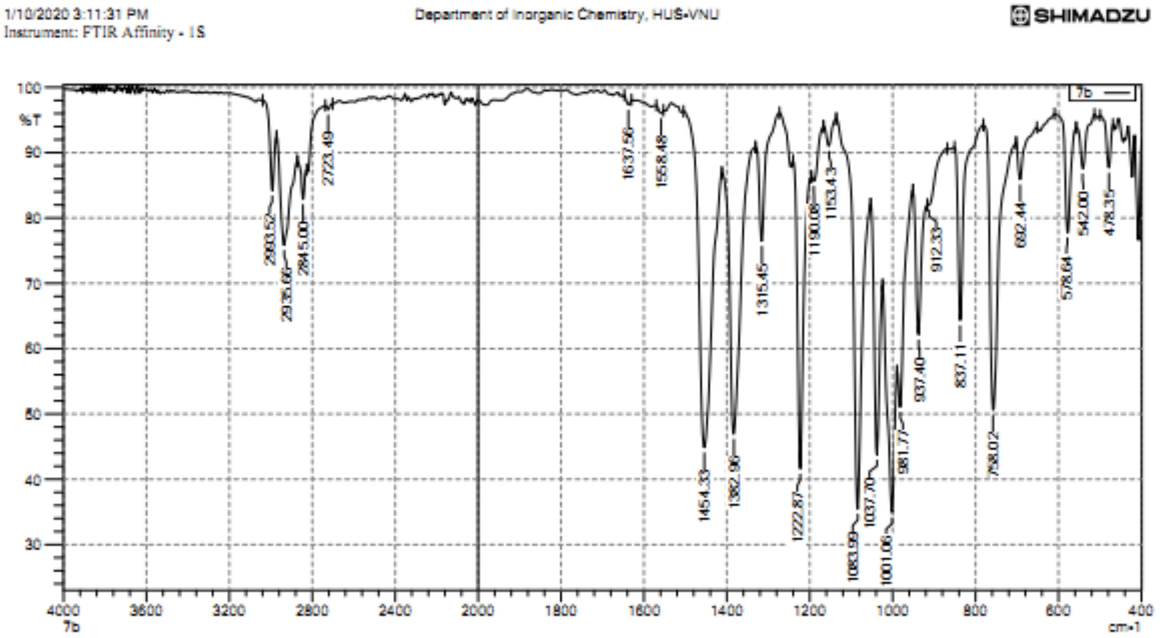

Comment:
File name: C:\Program Files\LabSolutions\IR\Data\Huu co\A Phong\Trang 2\7b.ispd
No of Scans: 20
Intensity Mode: %Transmittance
Min: 400 cm-1 Max: 4000 cm-1
Resolution: 4 [cm-1]
Atmosphere Correction: OFF

^{1}H NMR Spectrum (500 MHz, $CDCl_3$) of 1-allyl-2,5-dimethoxy-3,4,6-trimethylbenzene (**7b**)

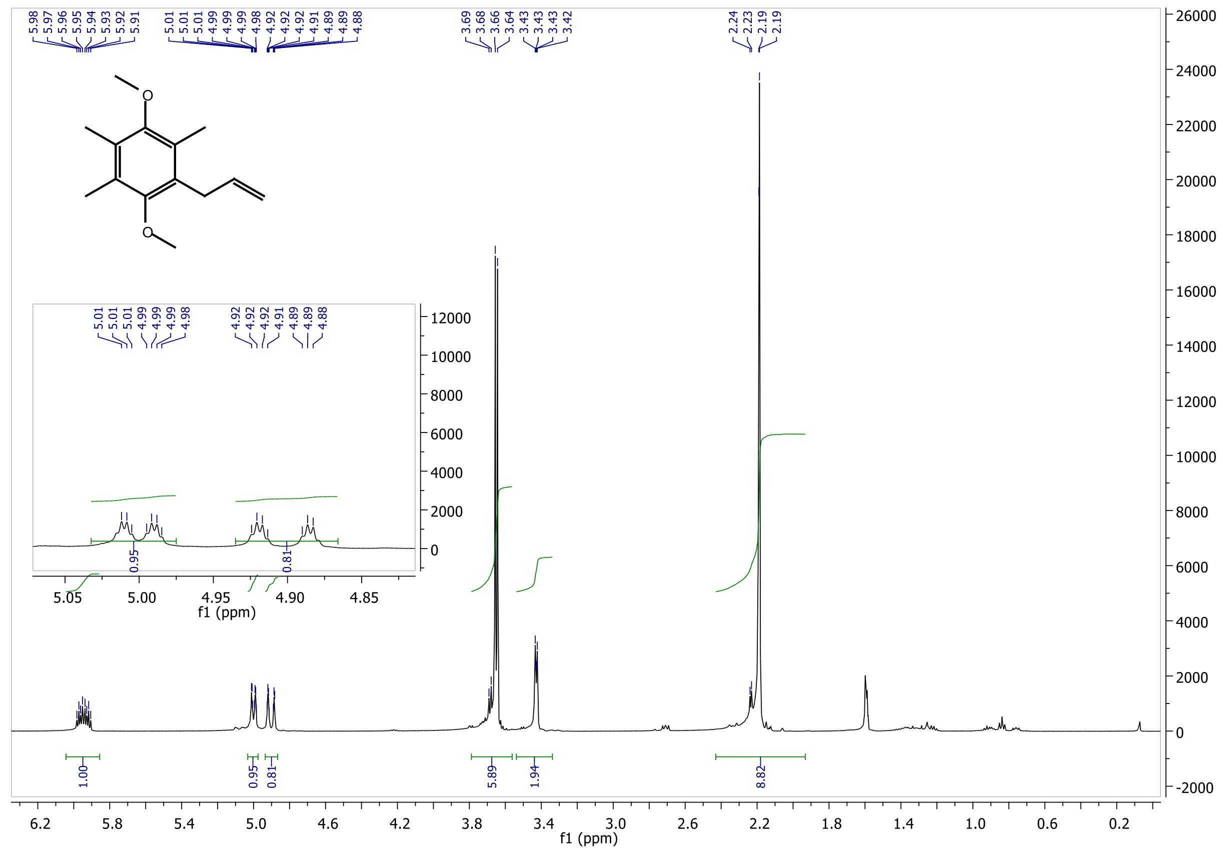

^{13}C NMR Spectrum (125 MHz, $CDCl_3$) of 1-allyl-2,5-dimethoxy-3,4,6-trimethylbenzene (**7b**)

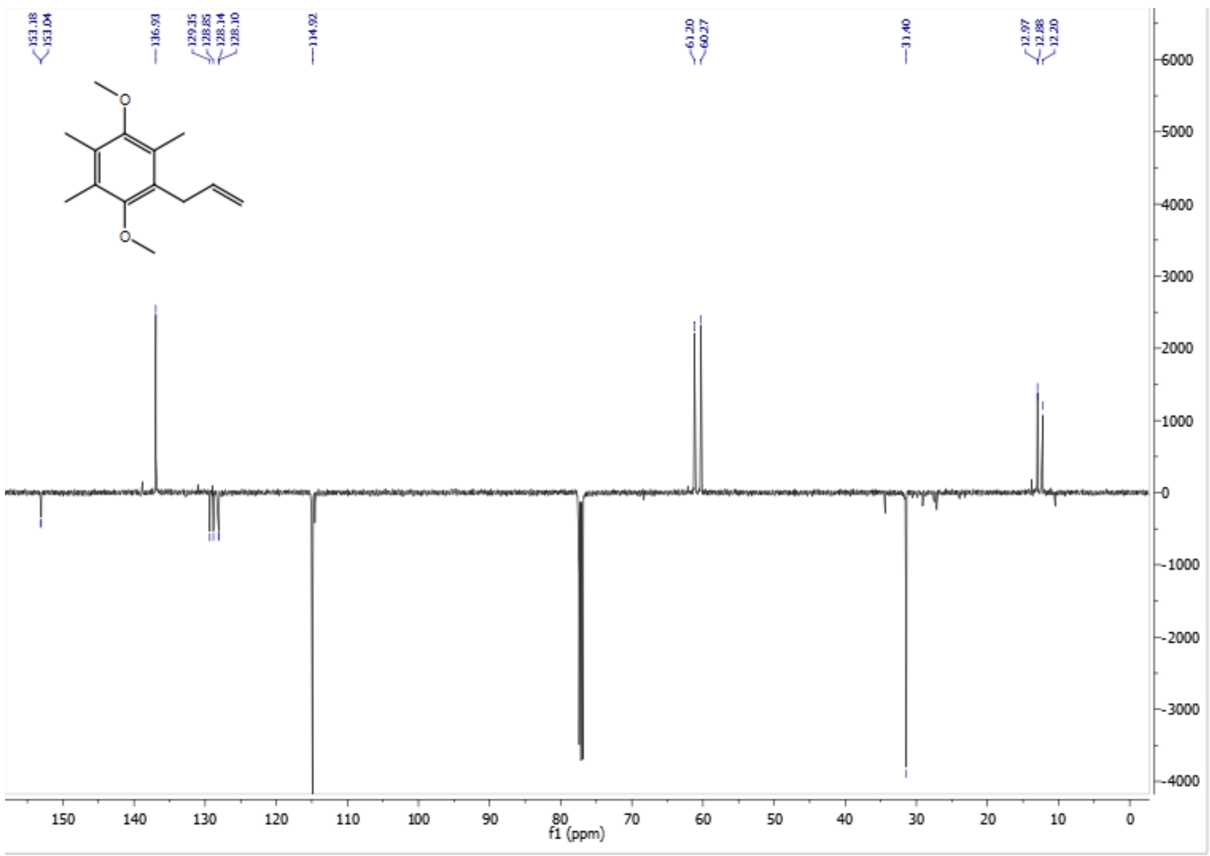

IR Spectrum of 1-allyl-2,3,4,5-tetramethoxy-6-methylbenzene (**7c**)

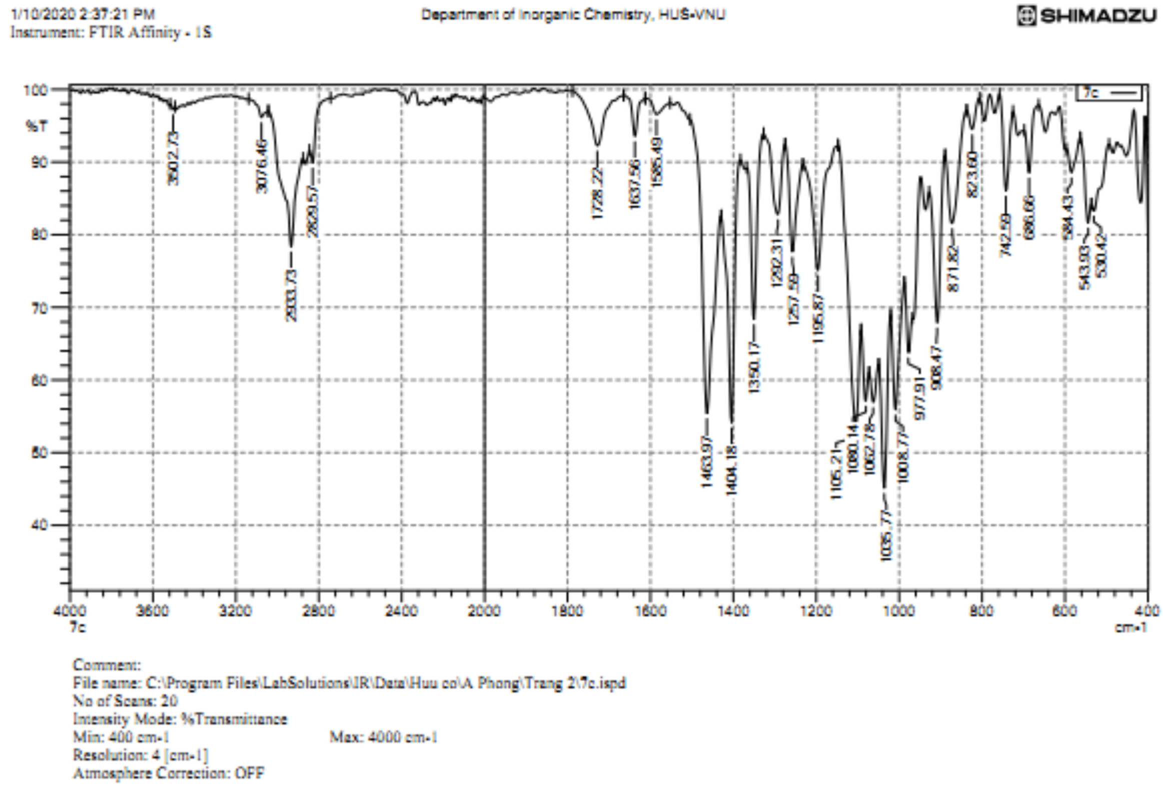

^{1}H NMR Spectrum (500 MHz, $CDCl_3$) of 1-allyl-2,3,4,5-tetramethoxy-6-methylbenzene (**7c**)

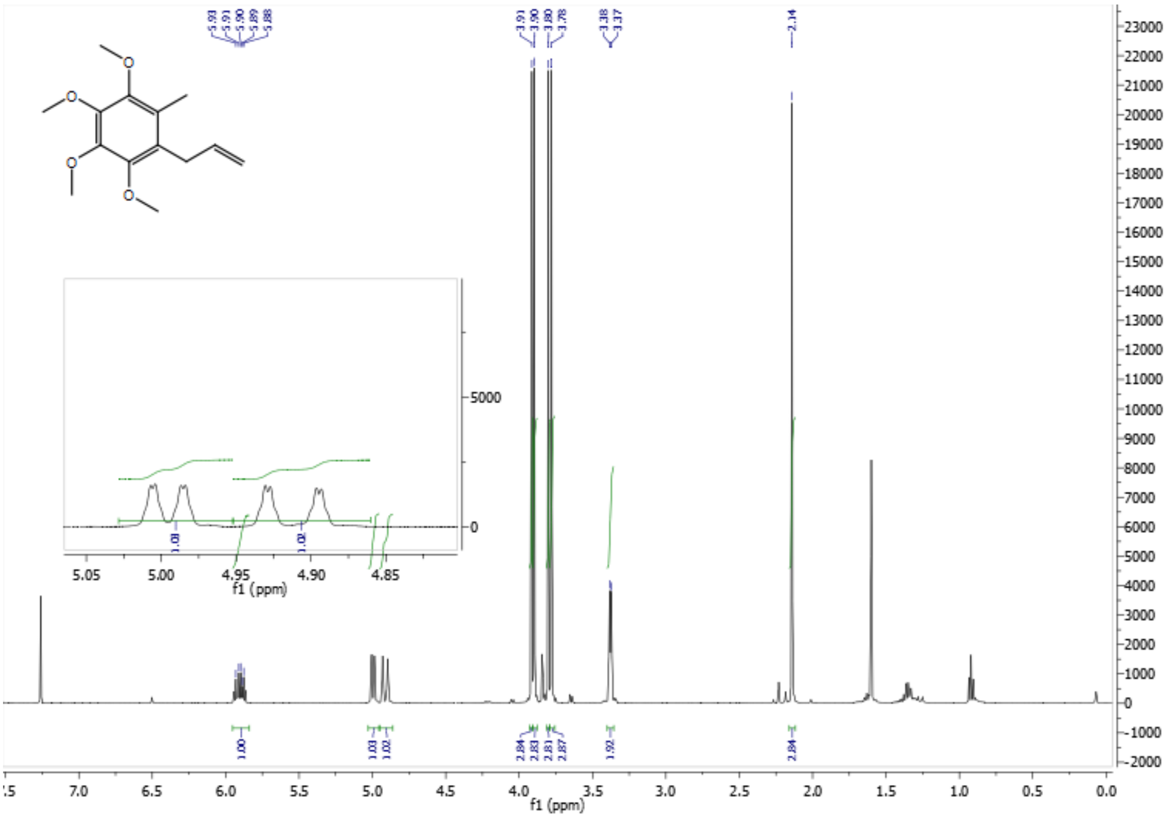

^{13}C NMR Spectrum (125 MHz, $CDCl_3$) of 1-allyl-2,3,4,5-tetramethoxy-6-methylbenzene (**7c**)

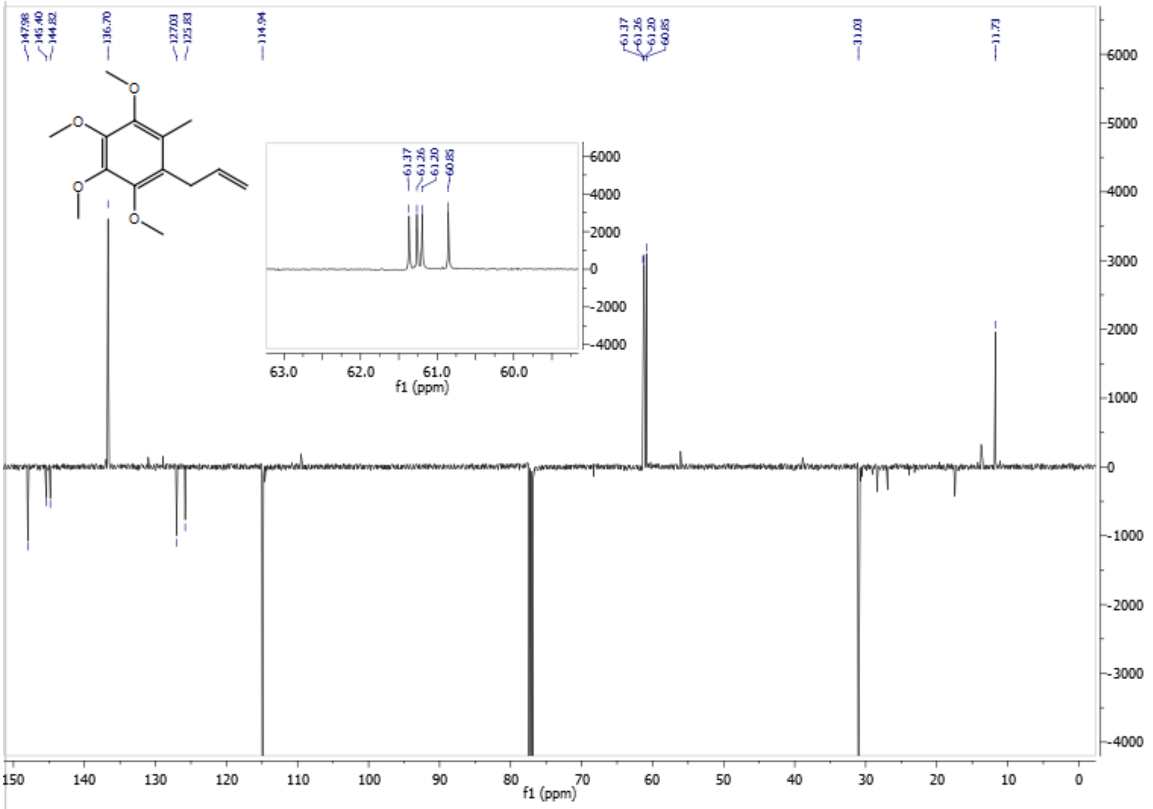

IR Spectrum (500 MHz, $CDCl_3$) of 2-allyl-3-methylnaphthalene-1,4-diyl diacetate (**7d**)

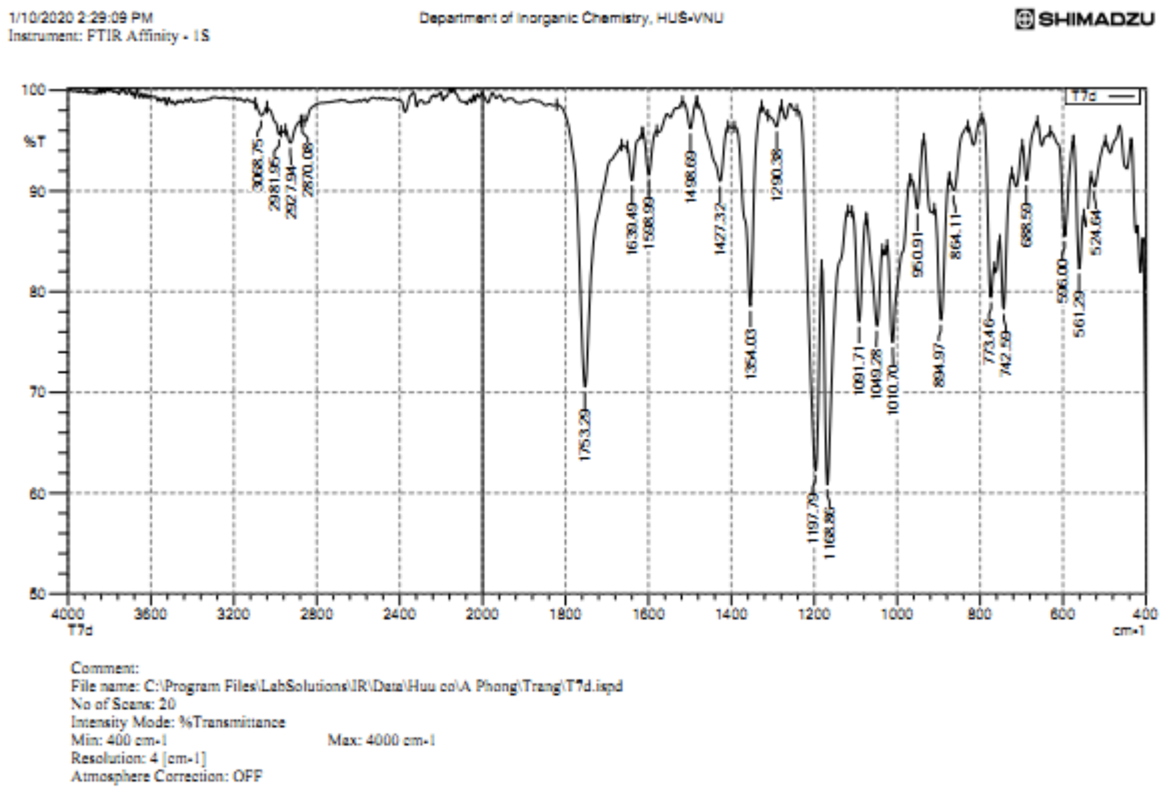

^{1}H NMR Spectrum (500 MHz, $CDCl_3$) of 2-allyl-3-methylnaphthalene-1,4-diyl diacetate (**7d**)

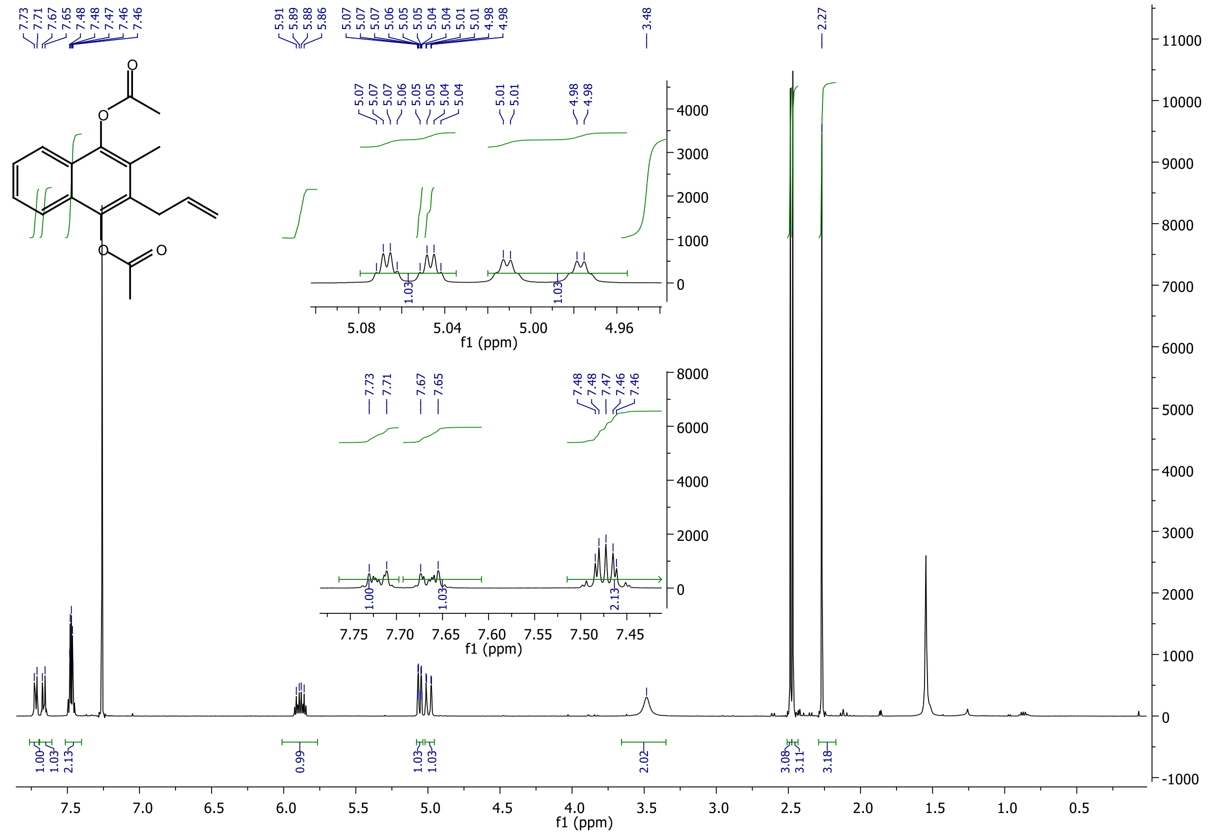

^{13}C NMR Spectrum (125 MHz, $CDCl_3$) of 2-allyl-3-methylnaphthalene-1,4-diyl diacetate (**7d**)

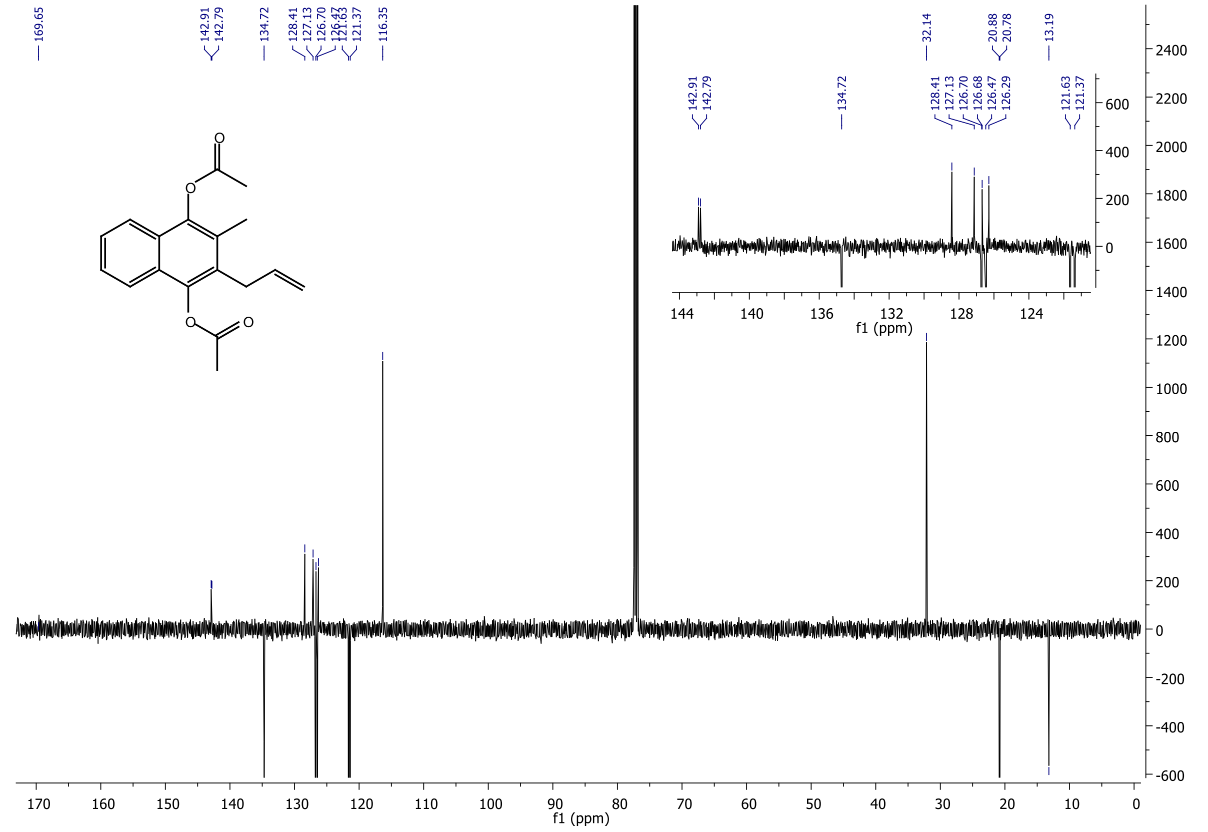

^{1}H NMR Spectrum (500 MHz, $CDCl_3$) of 2-allyl-1,4-dimethoxy-3-methylnaphthalene (**7e**)

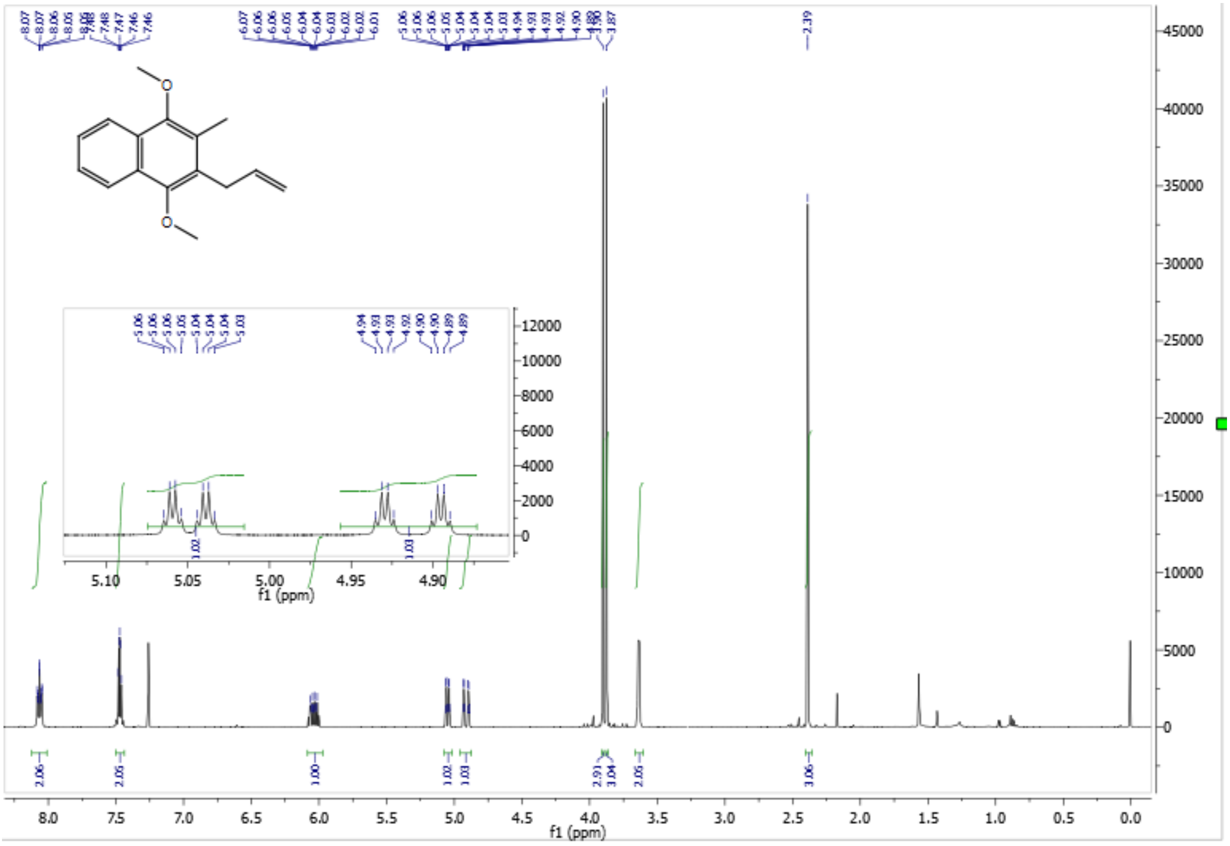

^{13}C NMR Spectrum (125 MHz, $CDCl_3$) of 2-allyl-1,4-dimethoxy-3-methylnaphthalene (**7e**)

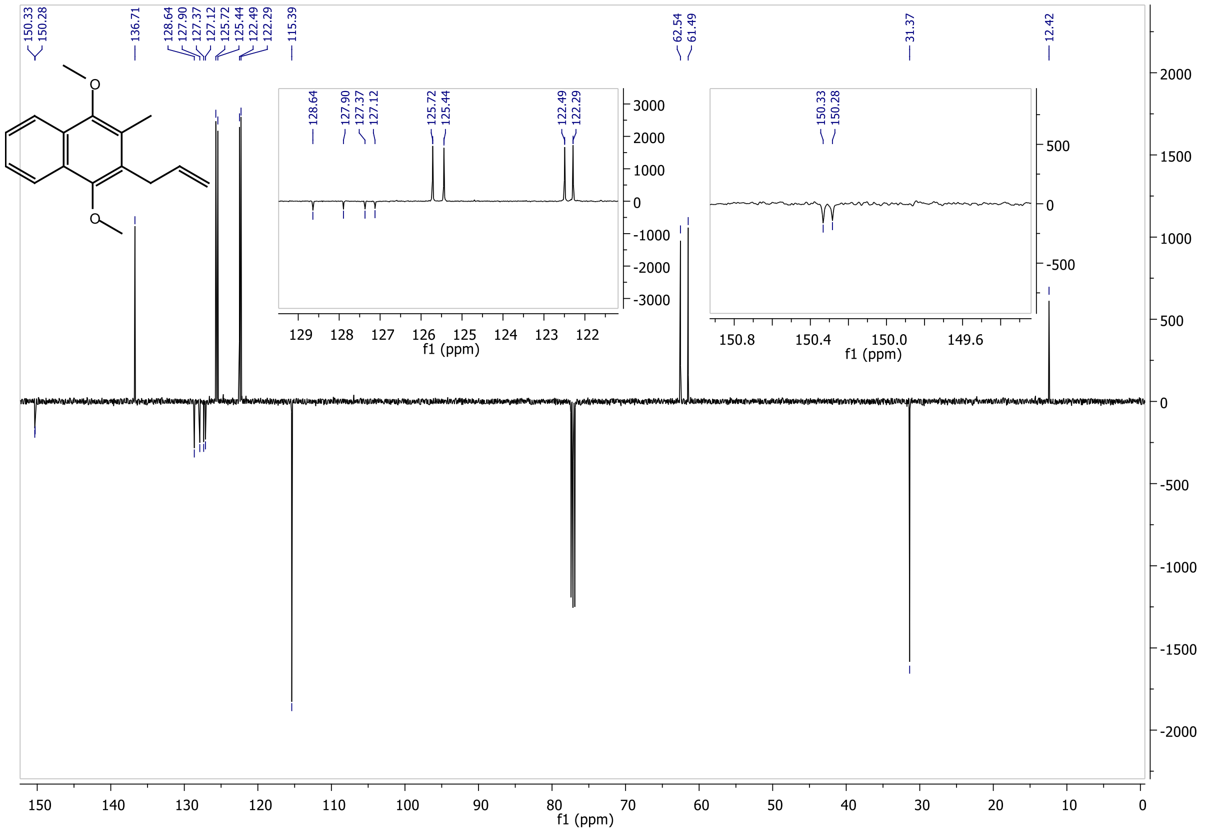

IR Spectrum of (*E*)-2-(4-methoxy-3-methyl-4-oxobut-2-en-1-yl)-3,5,6-trimethyl-1,4-phenylene diacetate (**8a**)

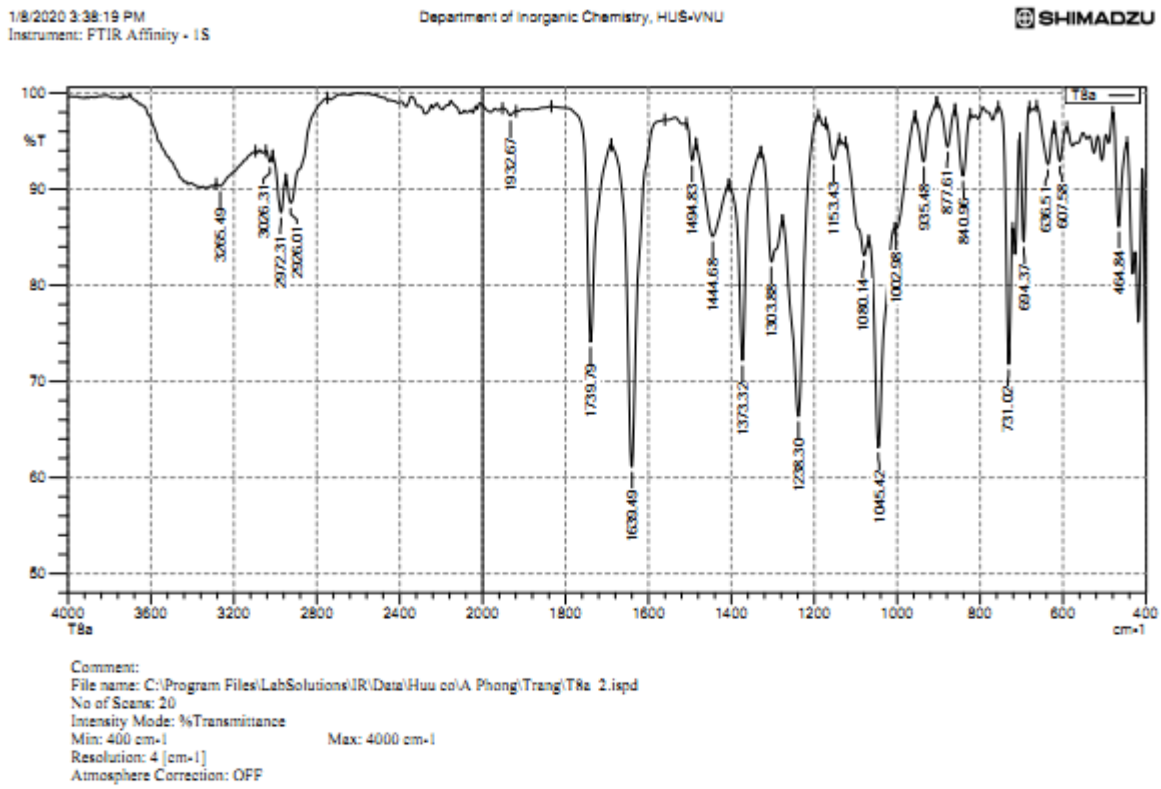

^{1}H NMR Spectrum (500 MHz, $CDCl_3$) of (*E*)-2-(4-methoxy-3-methyl-4-oxobut-2-en-1-yl)-3,5,6-trimethyl-1,4-phenylene diacetate (**8a**)

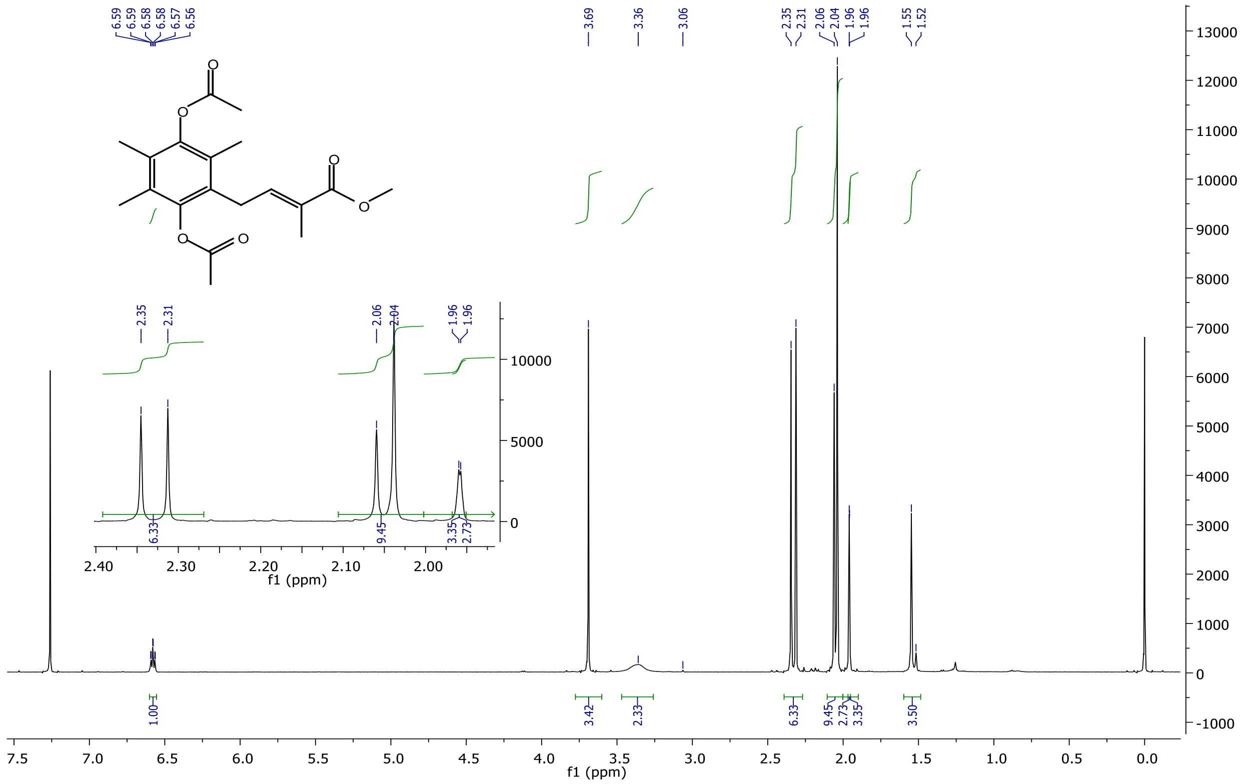

^{13}C NMR Spectrum (125 MHz, $CDCl_3$) of (*E*)-2-(4-methoxy-3-methyl-4-oxobut-2-en-1-yl)-3,5,6-trimethyl-1,4-phenylene diacetate (**8a**)

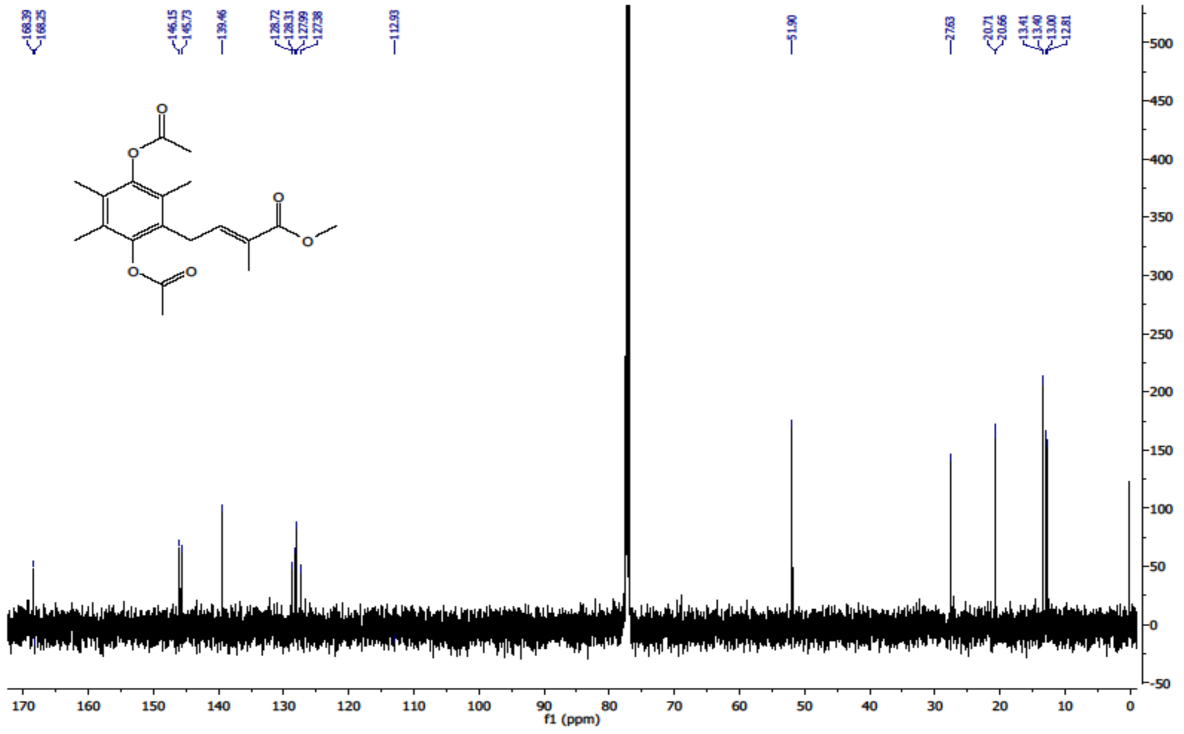

Noesy Spectrum (125 MHz, $CDCl_3$) of (*E*)-2-(4-methoxy-3-methyl-4-oxobut-2-en-1-yl)-3,5,6-trimethyl-1,4-phenylene diacetate (**8a**)

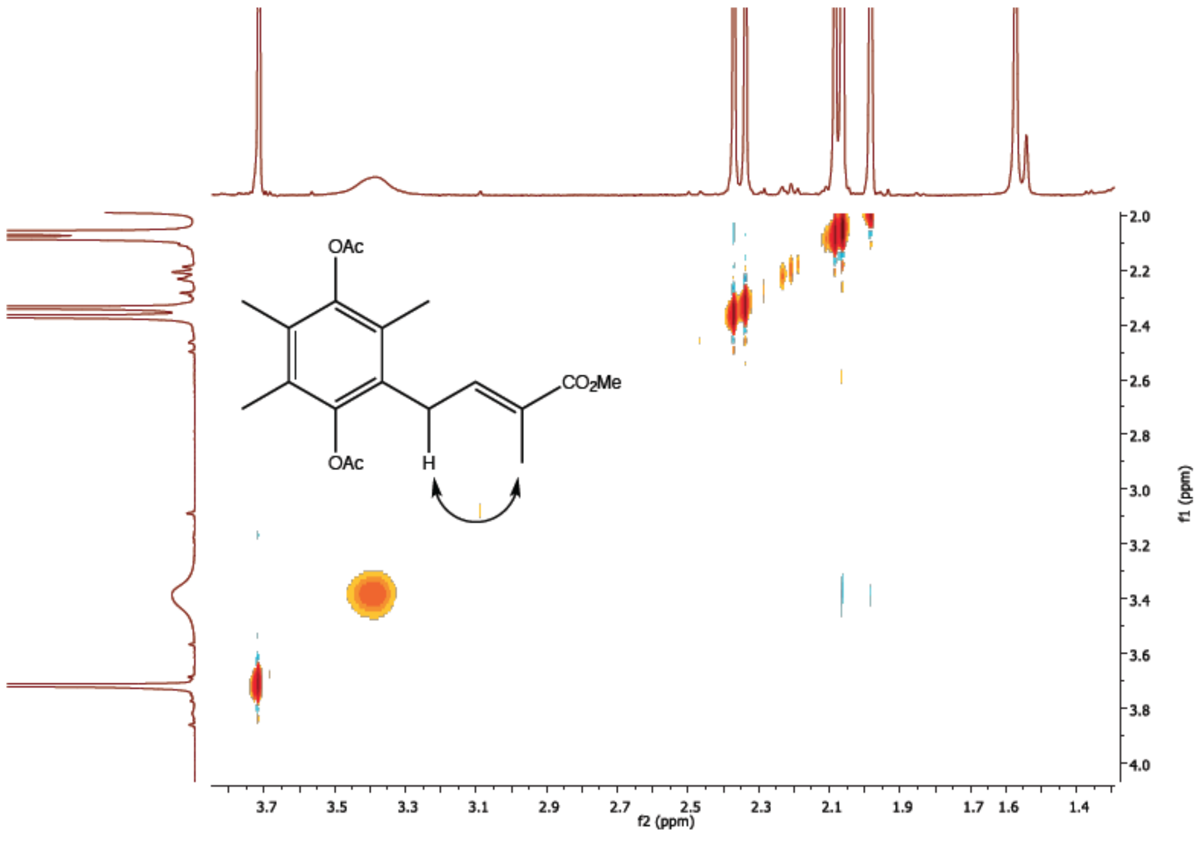

IR Spectrum of methyl (*E*)-4-(2,5-dimethoxy-3,4,6-trimethylphenyl)-2-methylbut-2-enoate (**8b**)

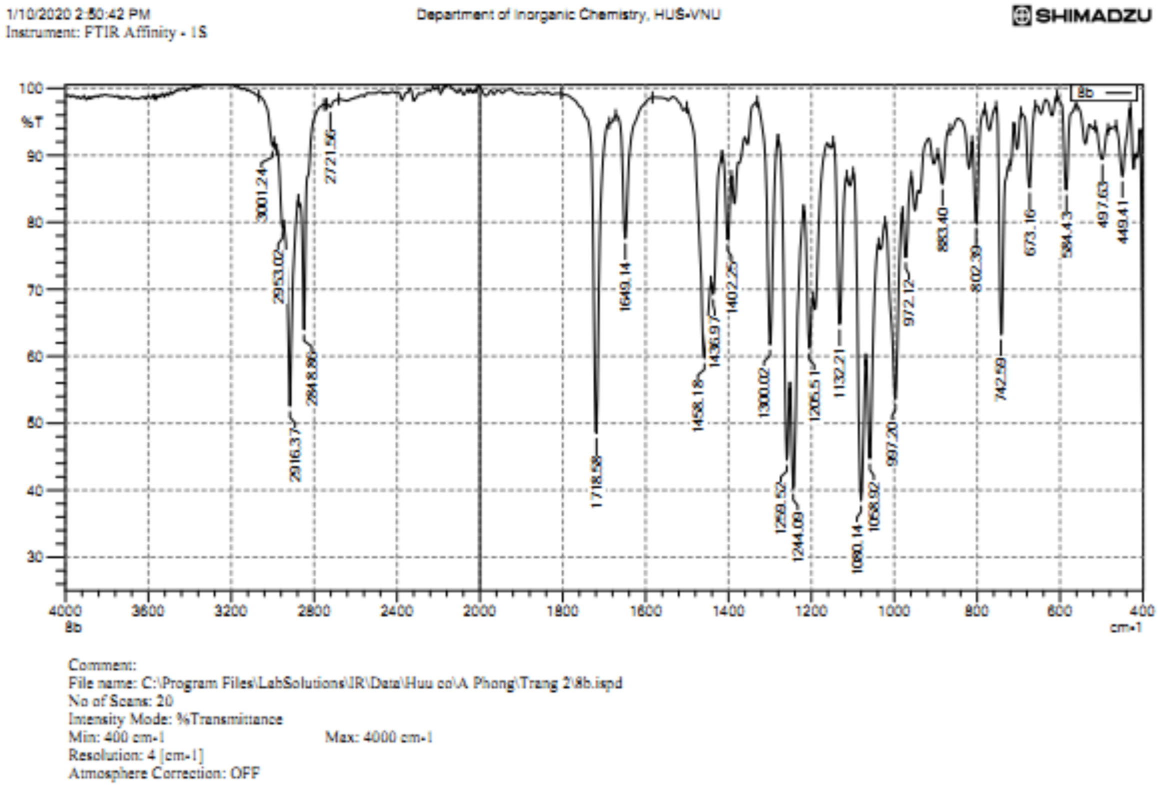

^{1}H NMR Spectrum (500 MHz, $CDCl_3$) of methyl (*E*)-4-(2,5-dimethoxy-3,4,6-trimethylphenyl)-2-methylbut-2-enoate (**8b**)

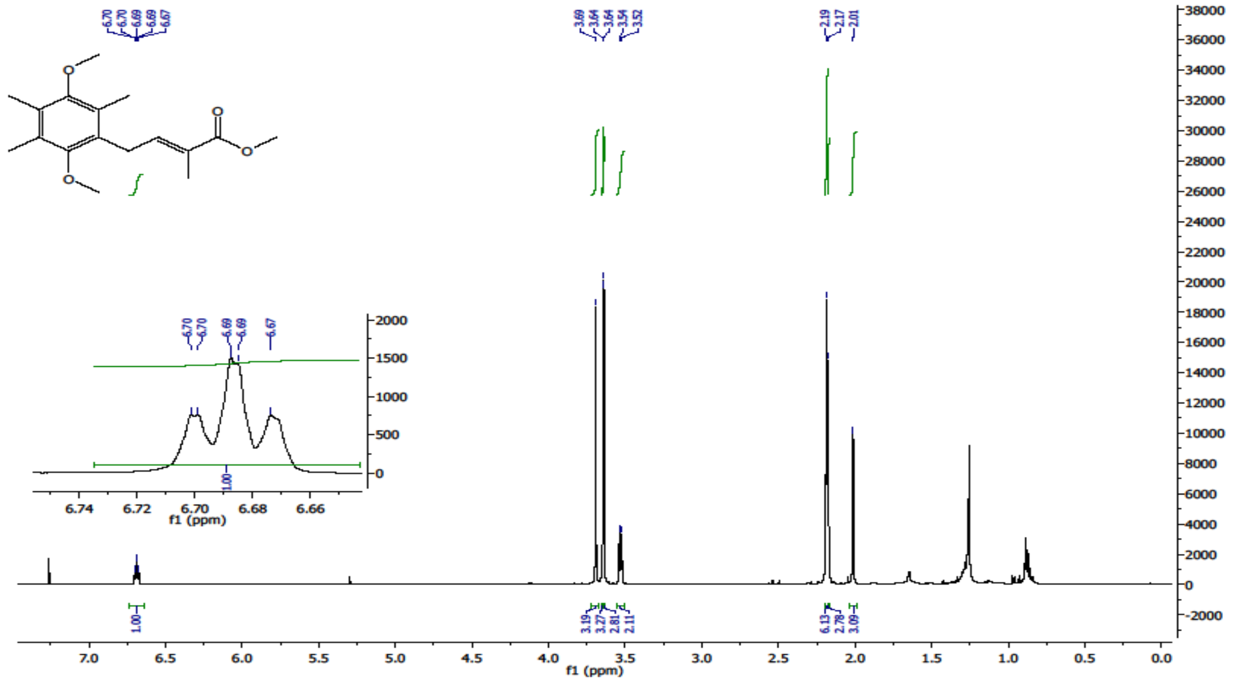

^{13}C NMR Spectrum (125 MHz, $CDCl_3$) of methyl (*E*)-4-(2,5-dimethoxy-3,4,6-trimethylphenyl)-2-methylbut-2-enoate (**8b**)

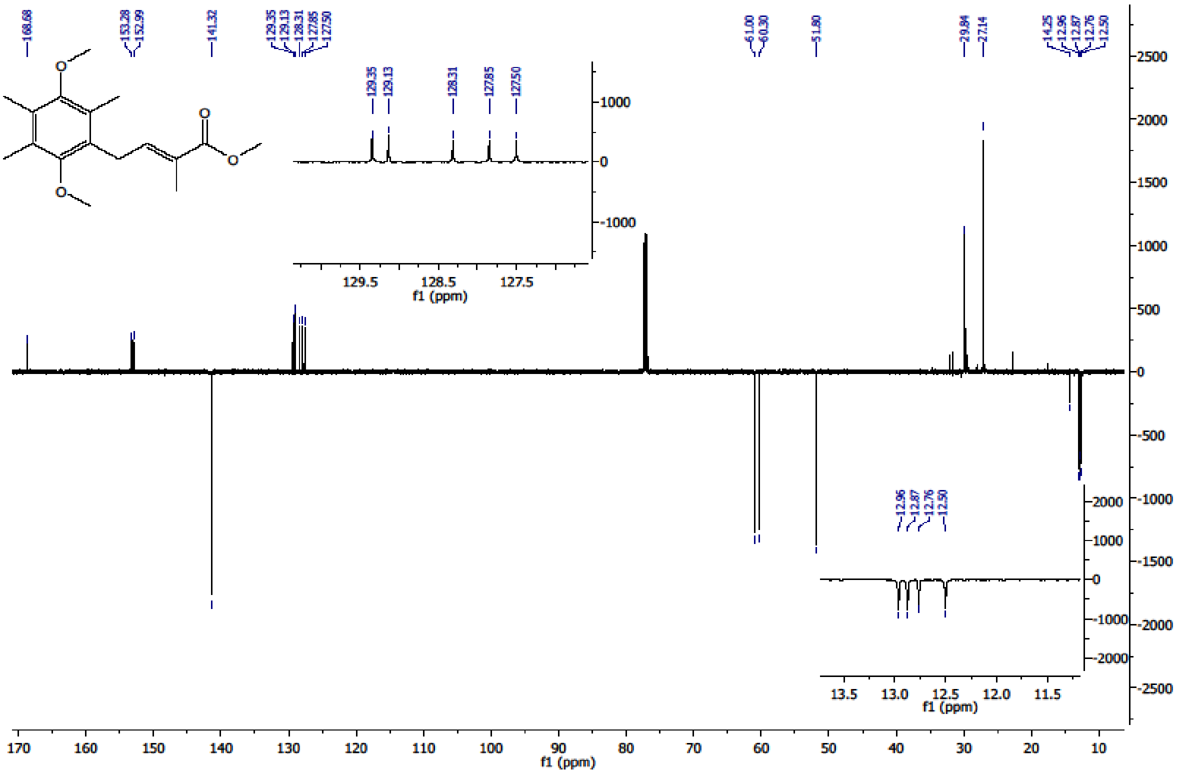

NOESY spectrum of methyl (*E*)-4-(2,5-dimethoxy-3,4,6-trimethylphenyl)-2-methylbut-2-enoate (**8b**)

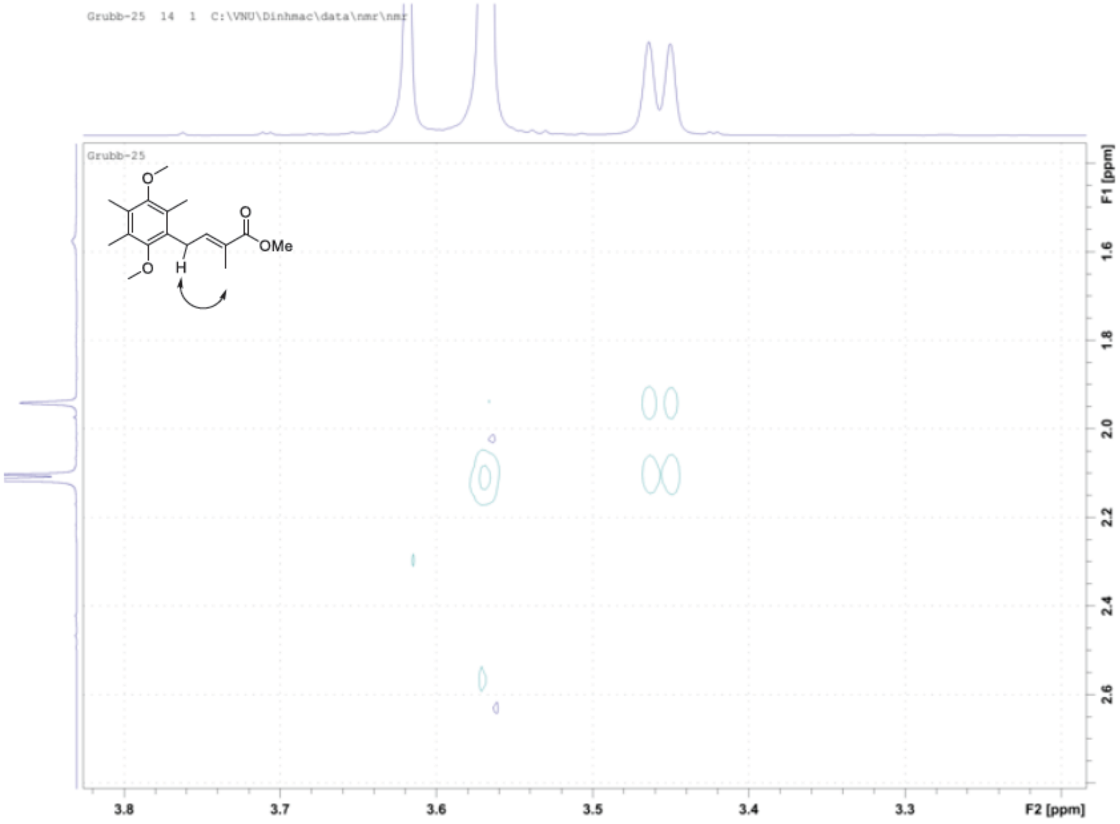

IR Spectrum of methyl (*E*)-2-methyl-4-(2,3,4,5-tetramethoxy-6-methylphenyl)but-2-enoate (**8c**)

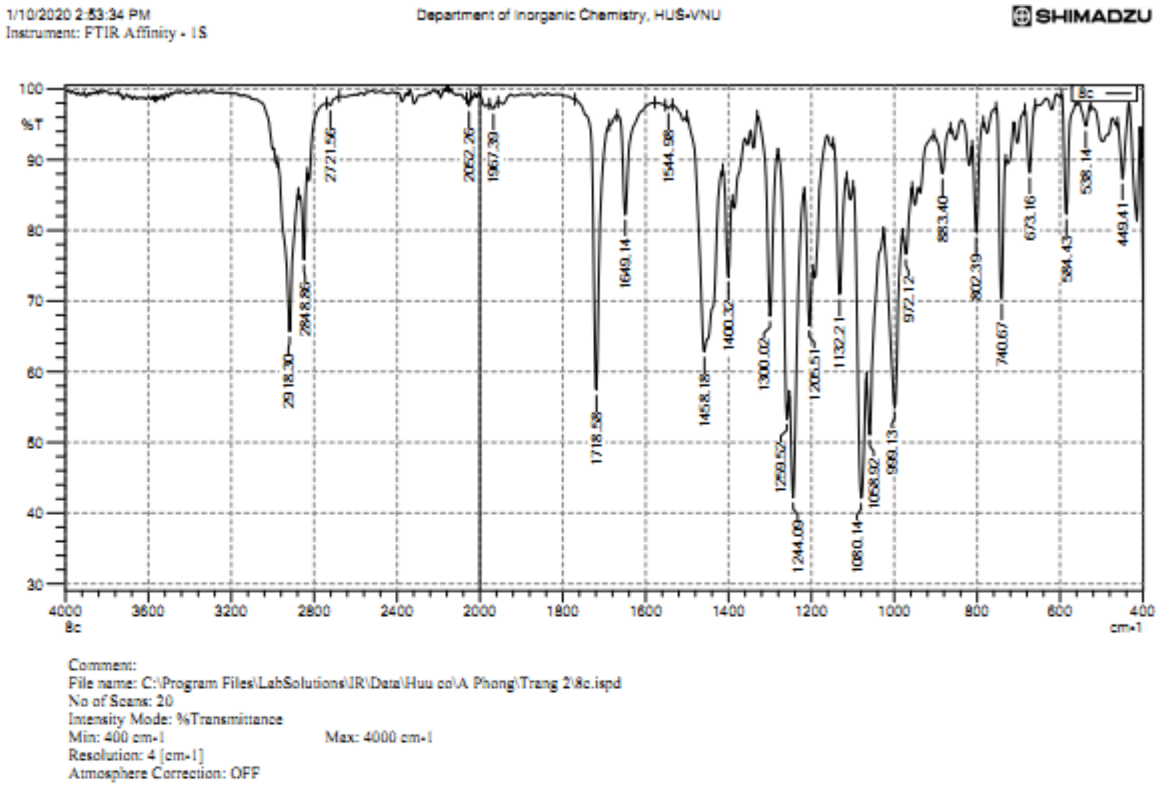

^{1}H NMR Spectrum (500 MHz, $CDCl_3$) of methyl (*E*)-2-methyl-4-(2,3,4,5-tetramethoxy-6-methylphenyl)but-2-enoate (**8c**)

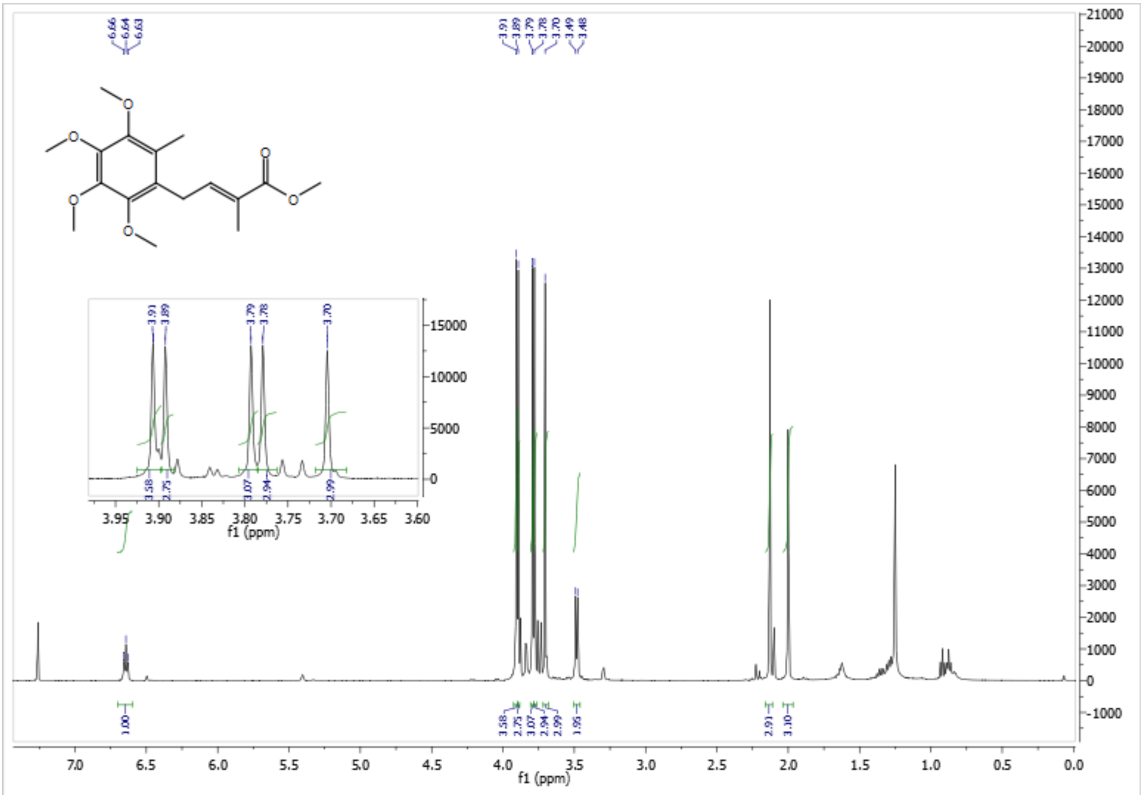

^{13}C NMR Spectrum (125 MHz, $CDCl_3$) of methyl (*E*)-2-methyl-4-(2,3,4,5-tetramethoxy-6-methylphenyl)but-2-enoate (**8c**)

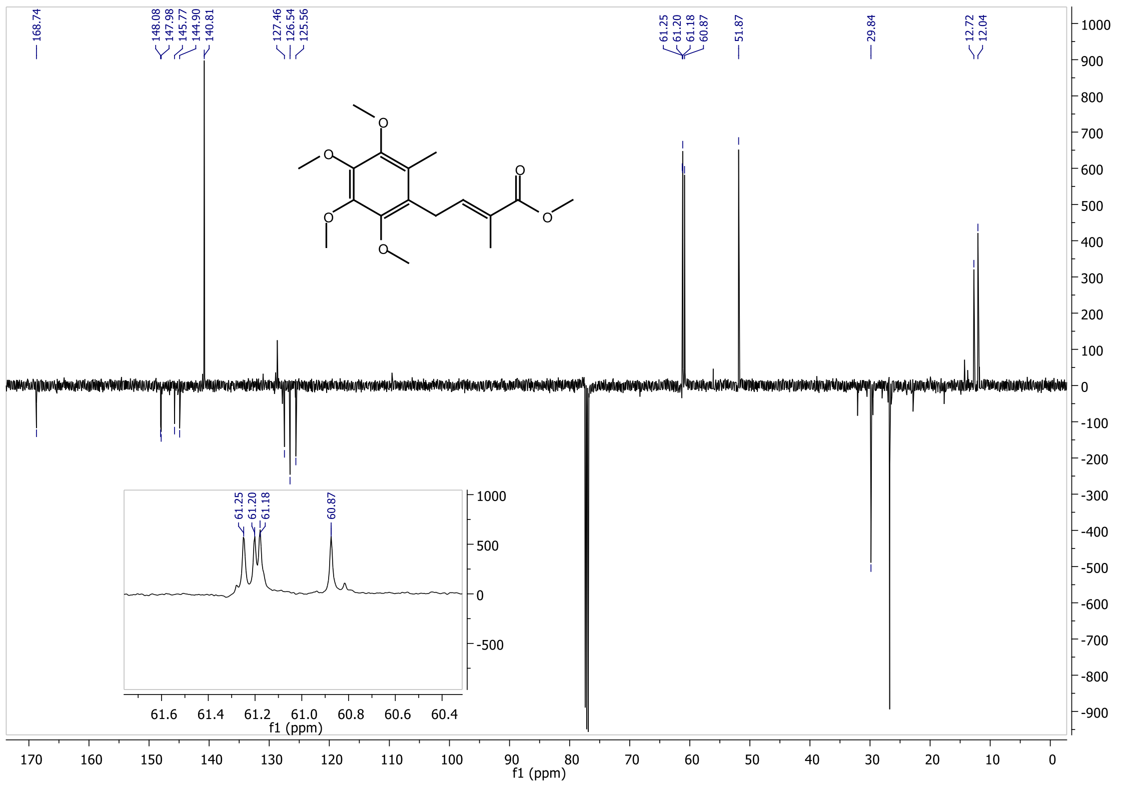

IR Spectrum of (*E*)-2-(4-methoxy-3-methyl-4-oxobut-2-en-1-yl)-3-methylnaphthalene-1,4-diyl diacetate (**8d**)

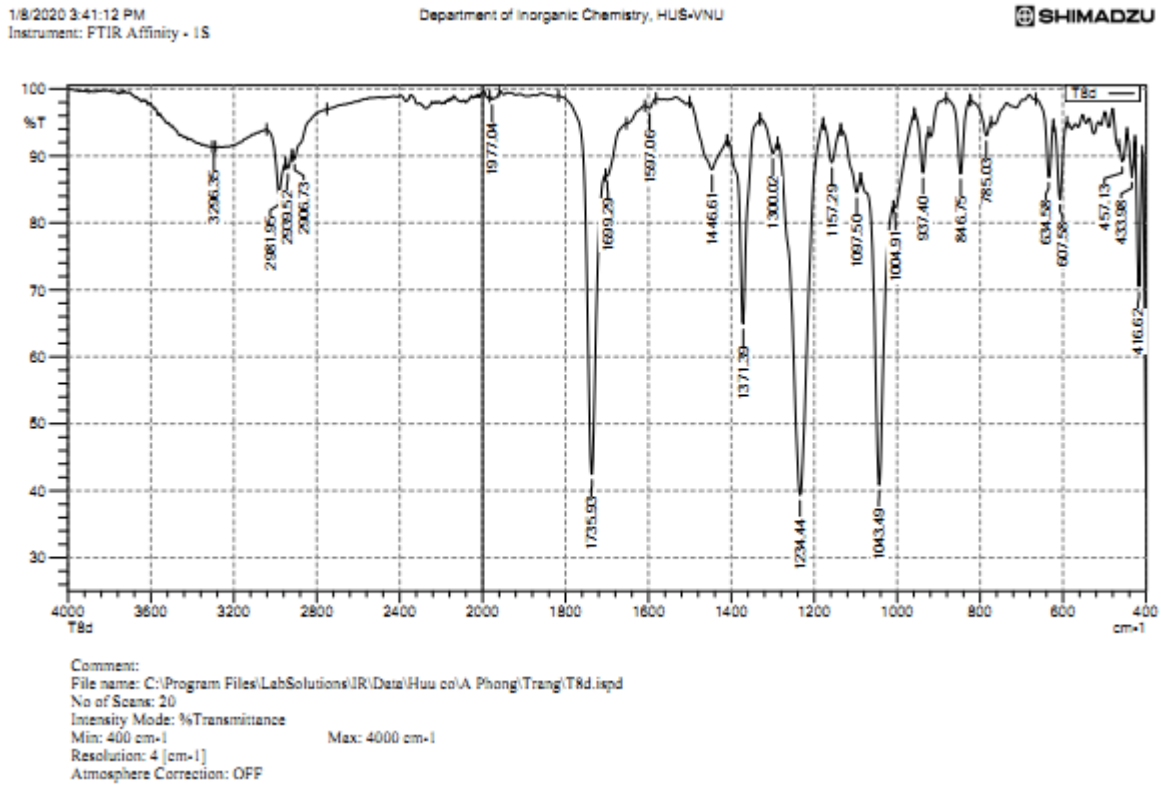

^{1}H NMR Spectrum (500 MHz, $CDCl_3$) of (*E*)-2-(4-methoxy-3-methyl-4-oxobut-2-en-1-yl)-3-methylnaphthalene-1,4-diyl diacetate (**8d**)

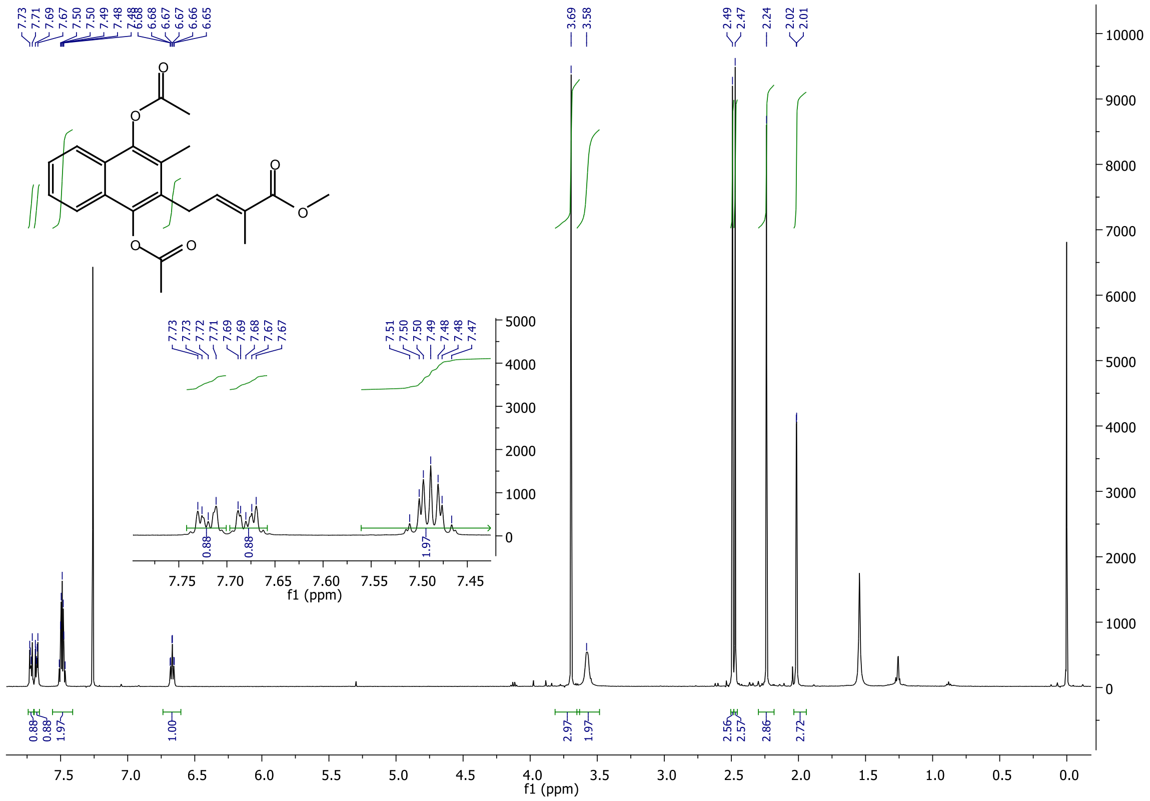

^{13}C NMR Spectrum (125 MHz, $CDCl_3$) of (*E*)-2-(4-methoxy-3-methyl-4-oxobut-2-en-1-yl)-3-methylnaphthalene-1,4-diyl diacetate (**8d**)

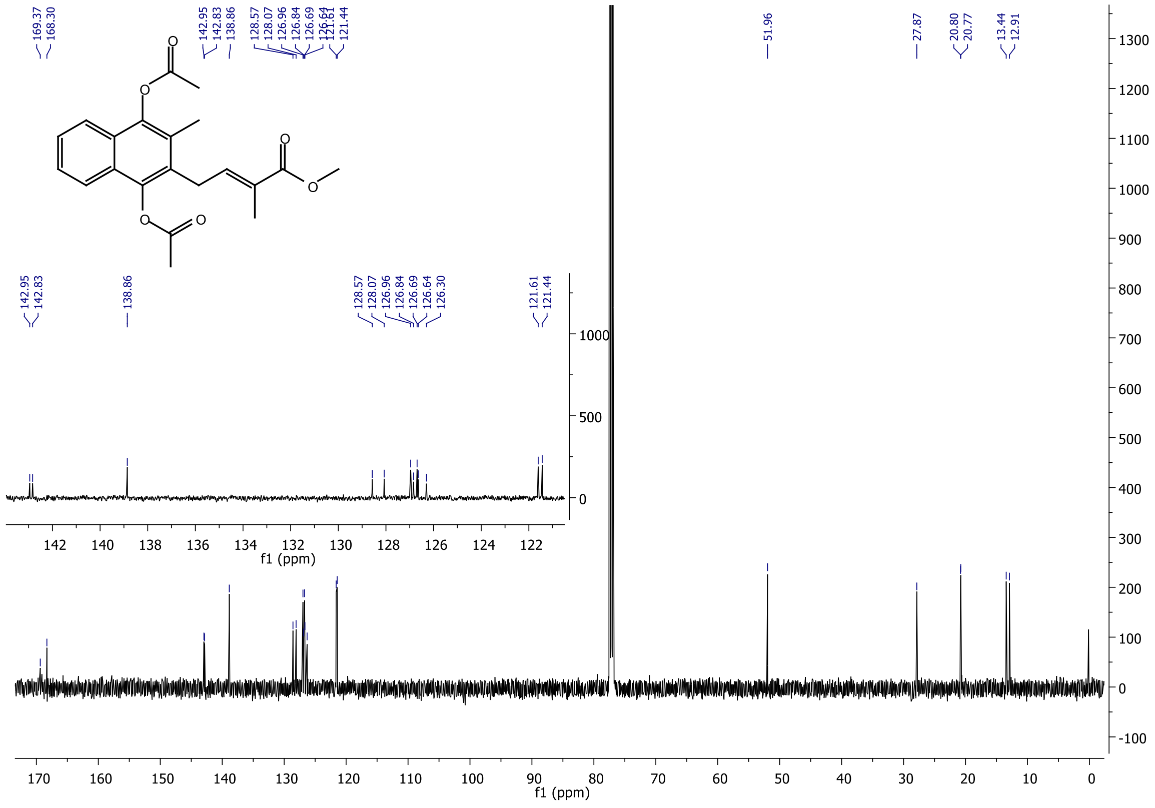

IR Spectrum of methyl (*E*)-4-(1,4-dimethoxy-3-methylnaphthalen-2-yl)-2-methylbut-2-enoate (**8e**)

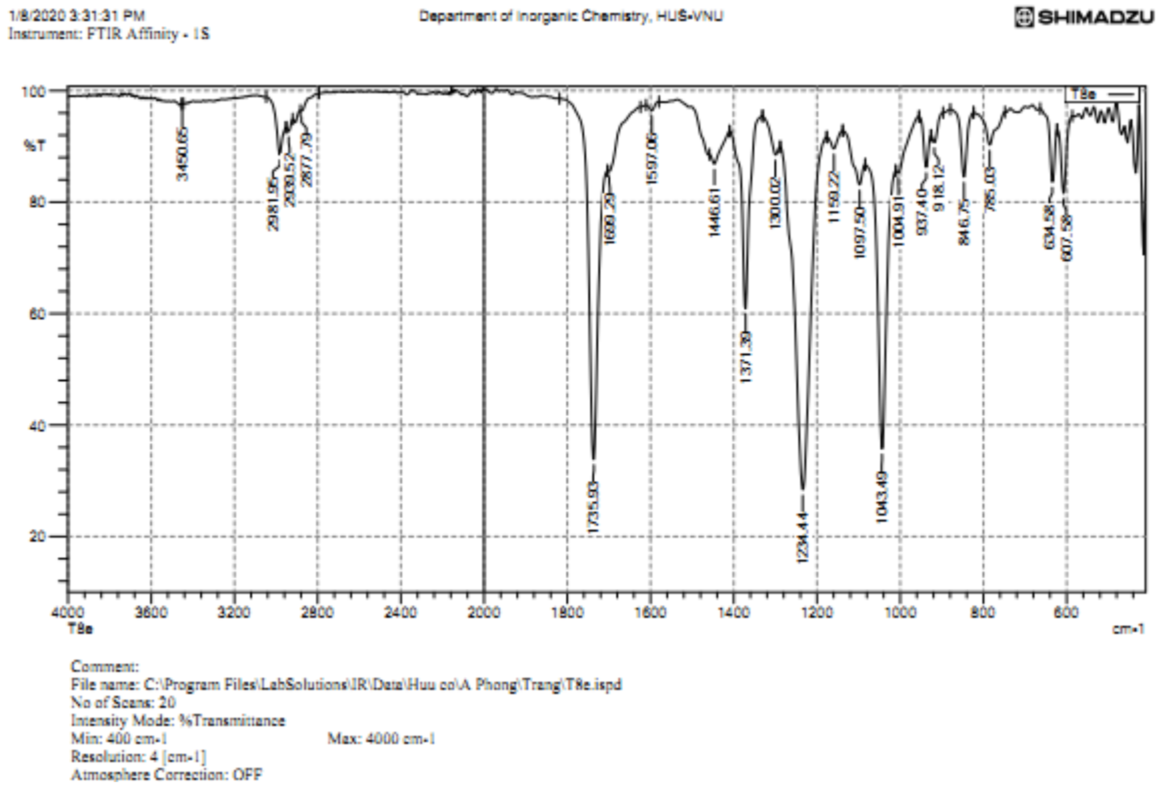

^{1}H NMR Spectrum (500 MHz, $CDCl_3$) of methyl (*E*)-4-(1,4-dimethoxy-3-methylnaphthalen-2-yl)-2-methylbut-2-enoate (**8e**)

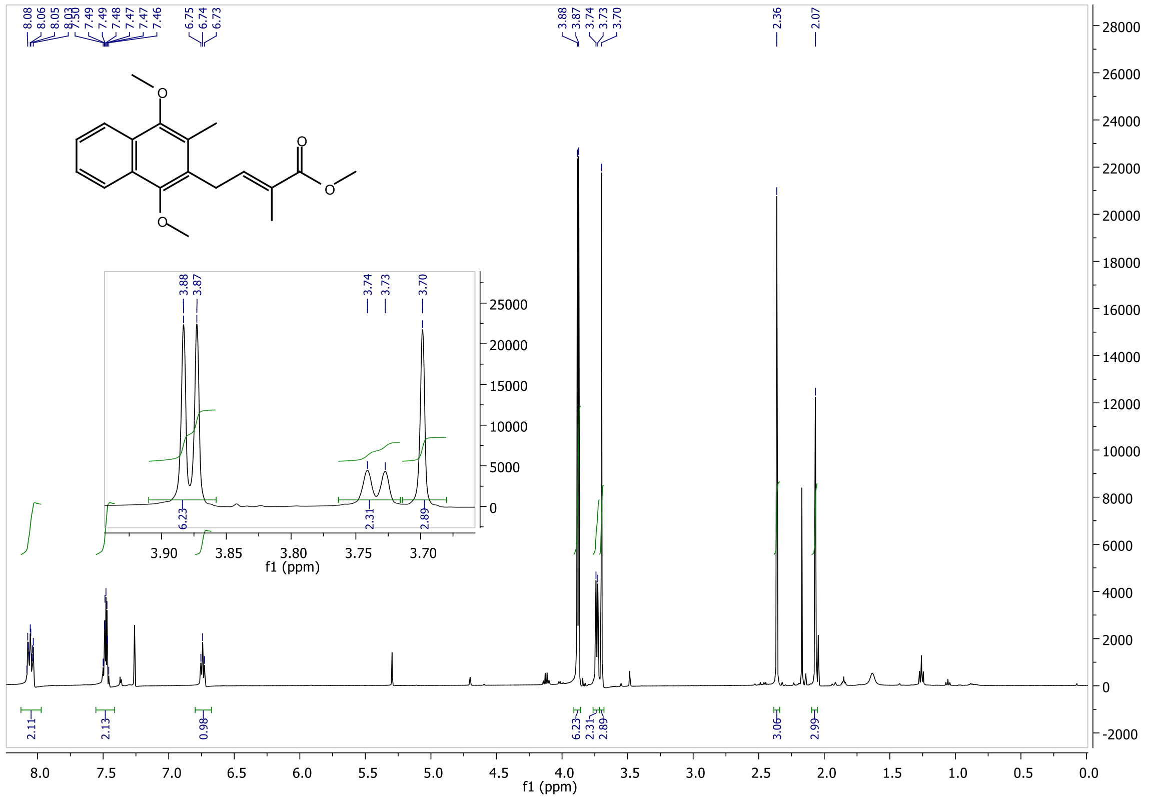

^{13}C NMR Spectrum (125 MHz, $CDCl_3$) of methyl (*E*)-4-(1,4-dimethoxy-3-methylnaphthalen-2-yl)-2-methylbut-2-enoate (**8e**)

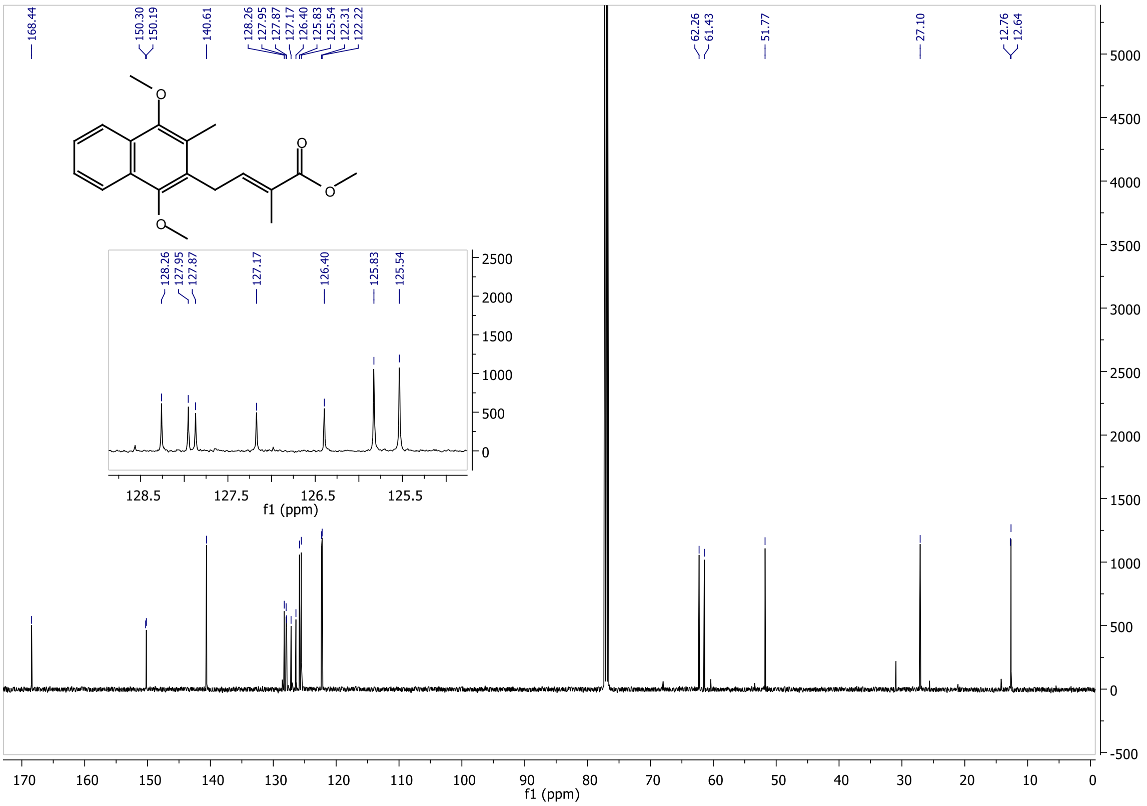

IR Spectrum of (*E*)-4-(2,5-dimethoxy-3,4,6-trimethylcyclohexa-1,5-dienyl)-2-methylbut-2-en-1-ol (**2b**)

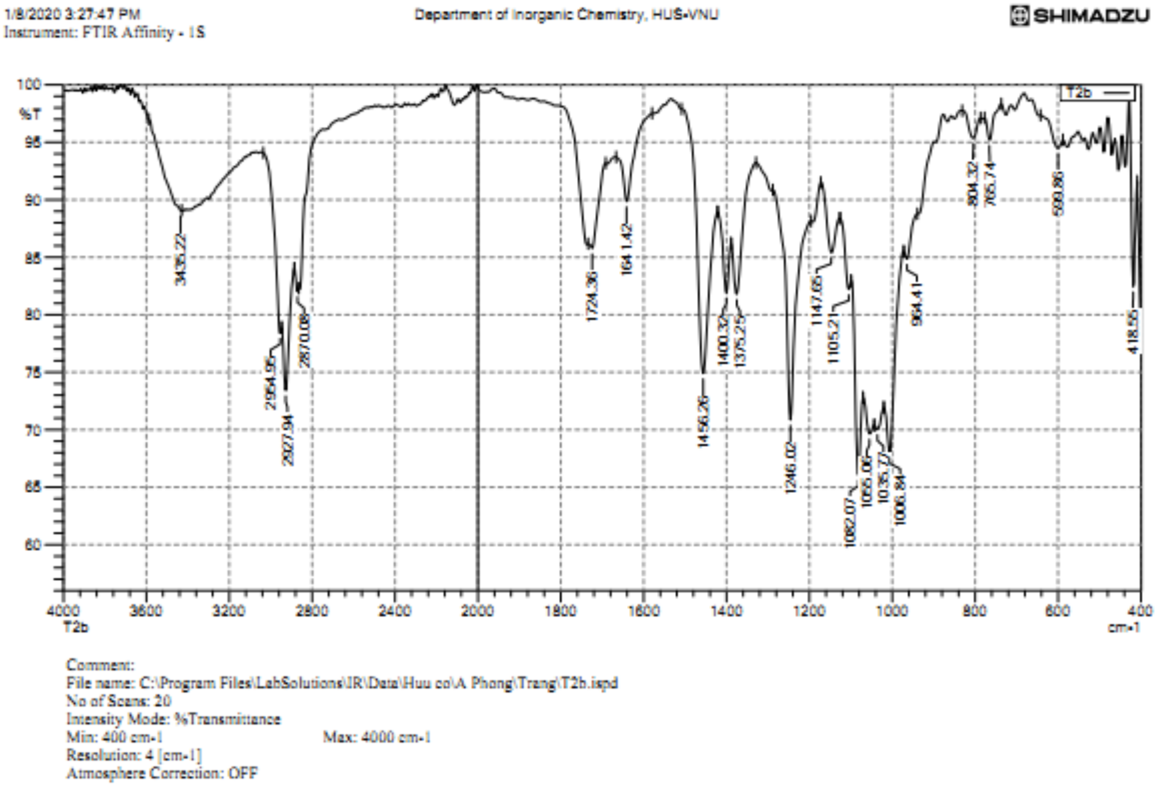

^{1}H NMR Spectrum (500 MHz, CDCl$_3$) of (*E*)-4-(2,5-dimethoxy-3,4,6-trimethylcyclohexa-1,5-dienyl)-2-methylbut-2-en-1-ol (**2b**)

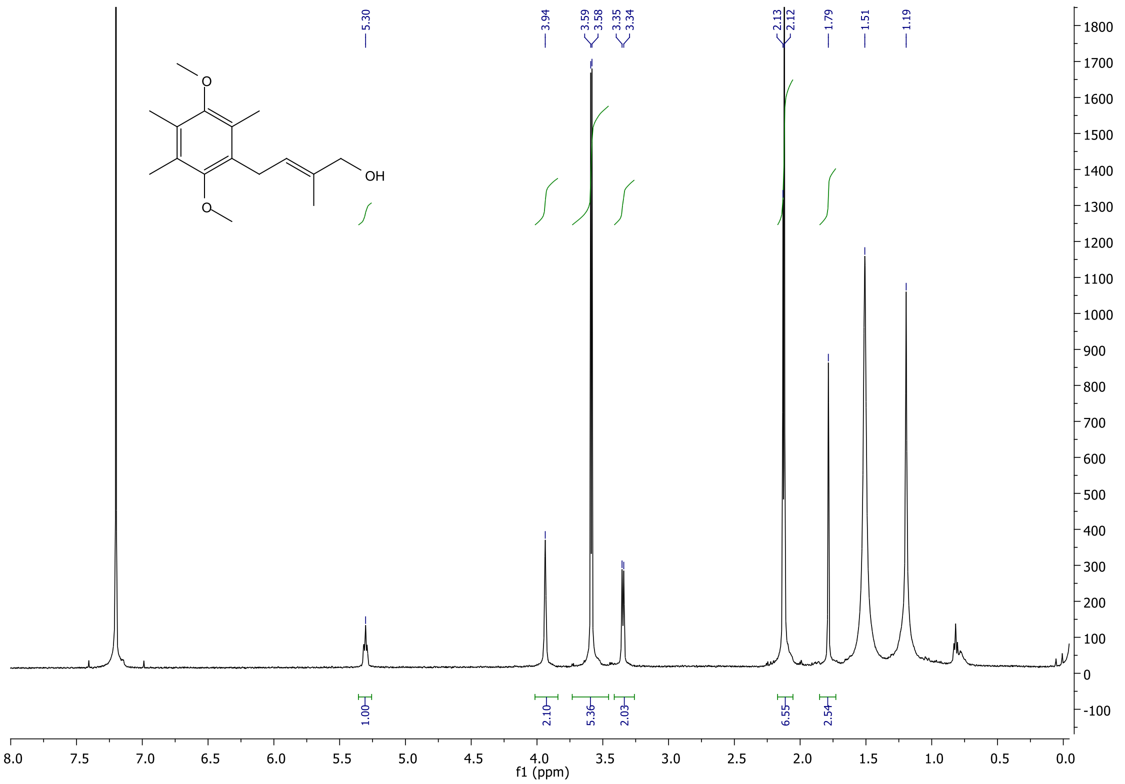

^{13}C NMR Spectrum (125 MHz, CDCl$_3$) of (*E*)-4-(2,5-dimethoxy-3,4,6-trimethylcyclohexa-1,5-dienyl)-2-methylbut-2-en-1-ol (**2b**)

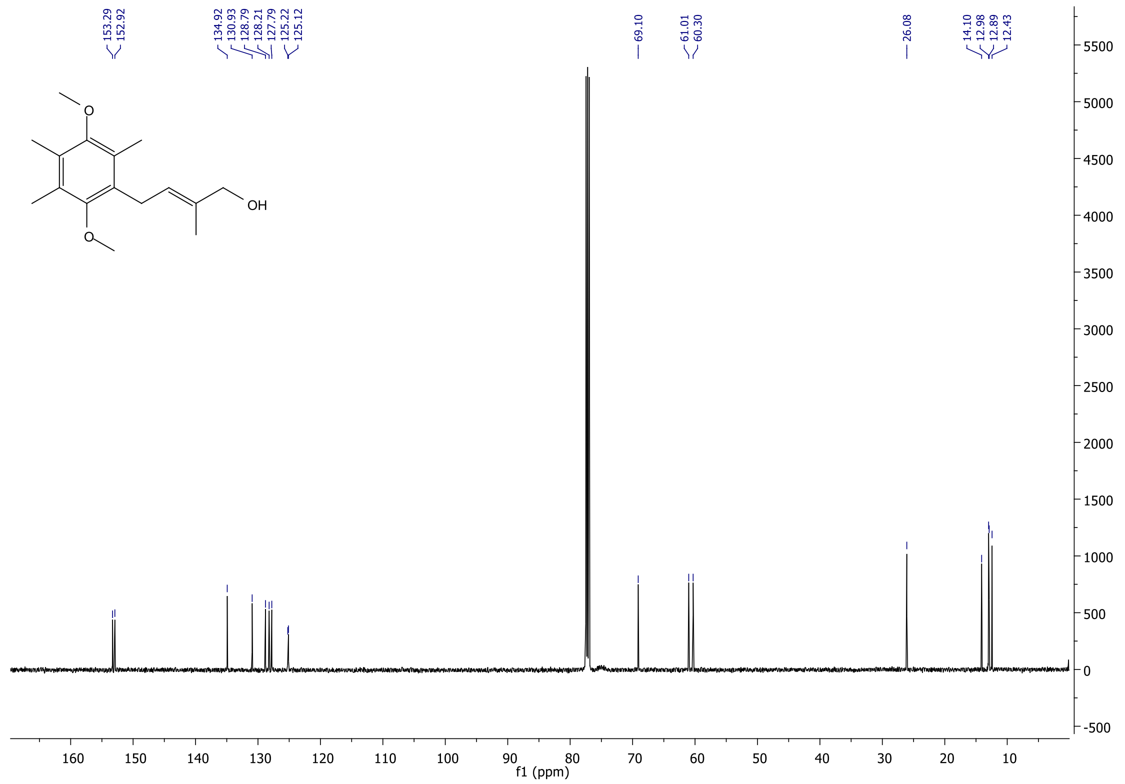

IR Spectrum of (*E*)-2-methyl-4-(2,3,4,5-tetramethoxy-6-methylphenyl)but-2-en-1-ol (**2c**)

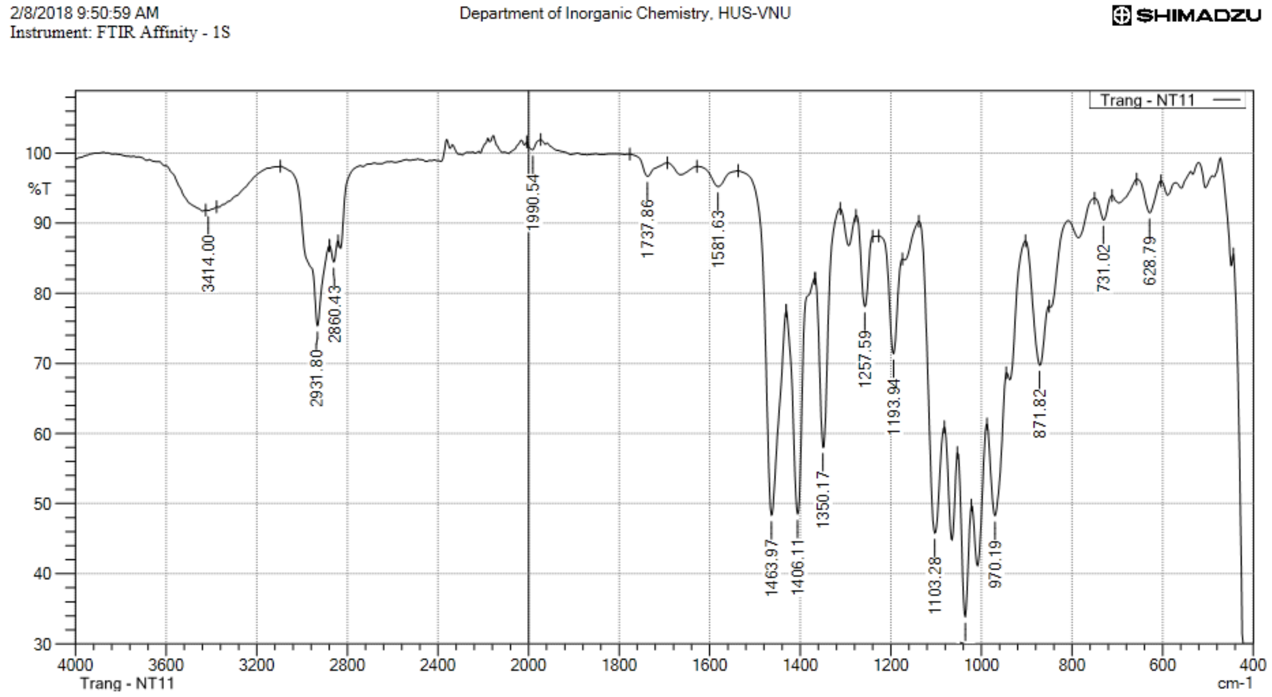

Spectrum (500 MHz, CDCl3) of methyl (*E*)-2-methyl-4-(2,3,4,5-tetramethoxy-6-methylphenyl)but-2-en-1-ol (**2c**)

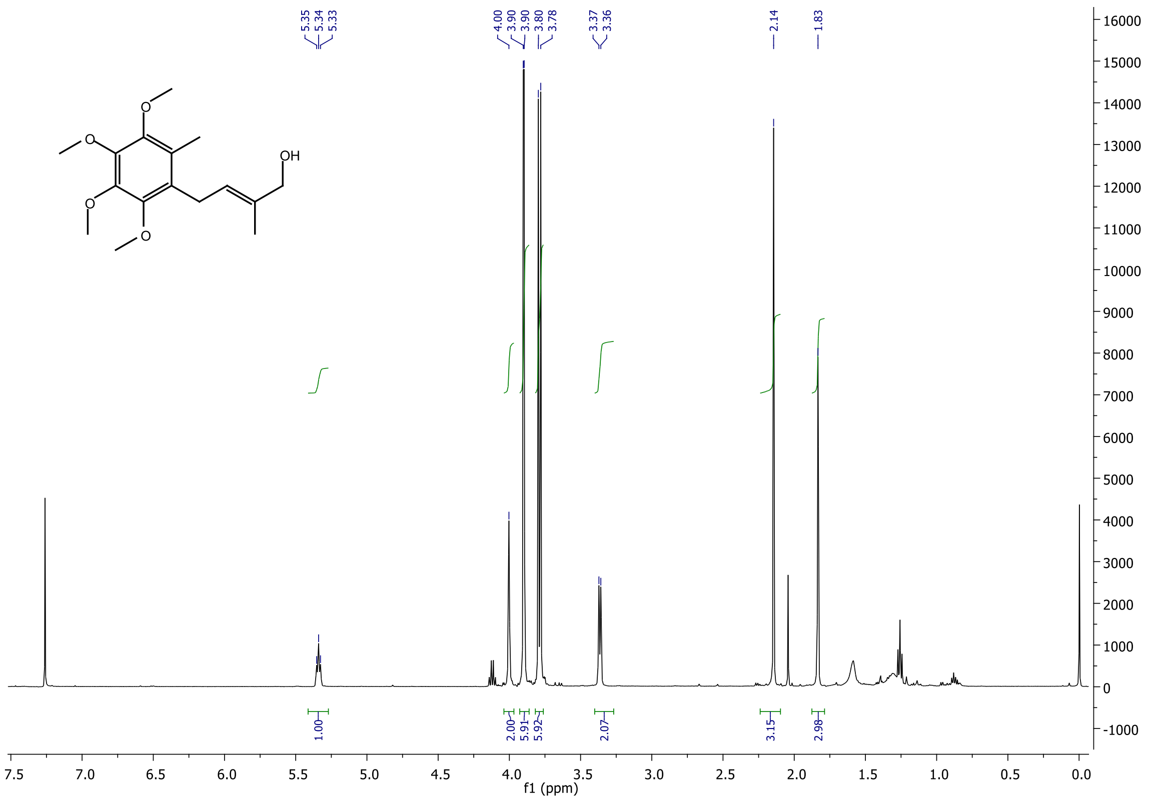

^{13}C NMR Spectrum (125 MHz, CDCl3) of (*E*)-2-methyl-4-(2,3,4,5-tetramethoxy-6-methylphenyl)but-2-en-1-ol (**2c**)

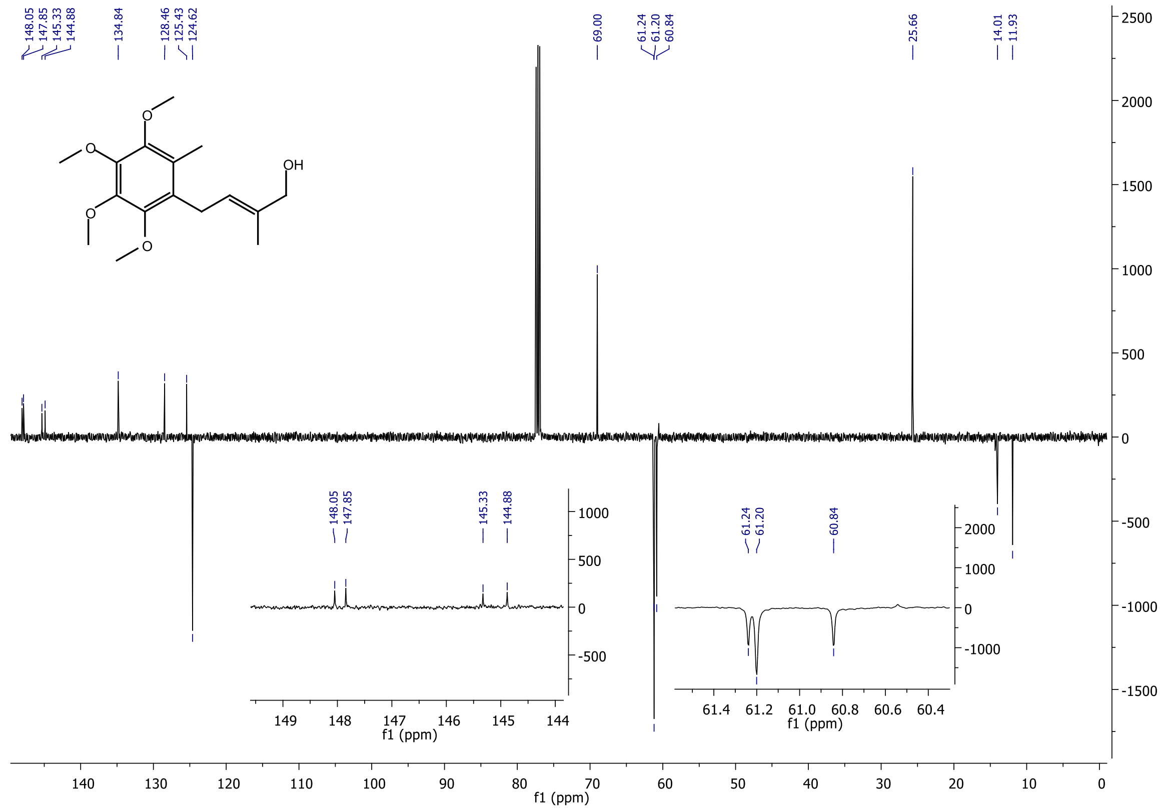

IR Spectrum of (*E*)-4-(1,4-dimethoxy-3-methylnaphthalen-2-yl)-2-methylbut-2-en-1-ol (**2e**)

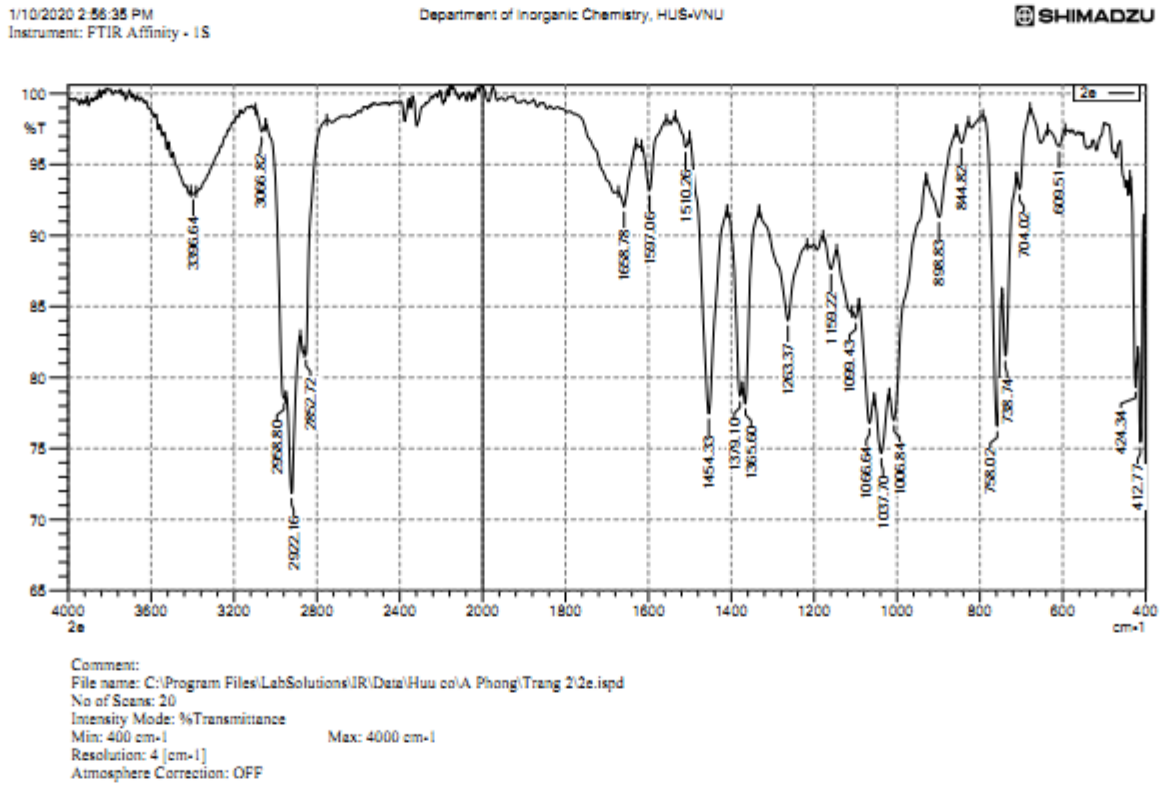

^{1}H NMR Spectrum (500 MHz, $CDCl_3$) of (*E*)-4-(1,4-dimethoxy-3-methylnaphthalen-2-yl)-2-methylbut-2-en-1-ol (**2e**)

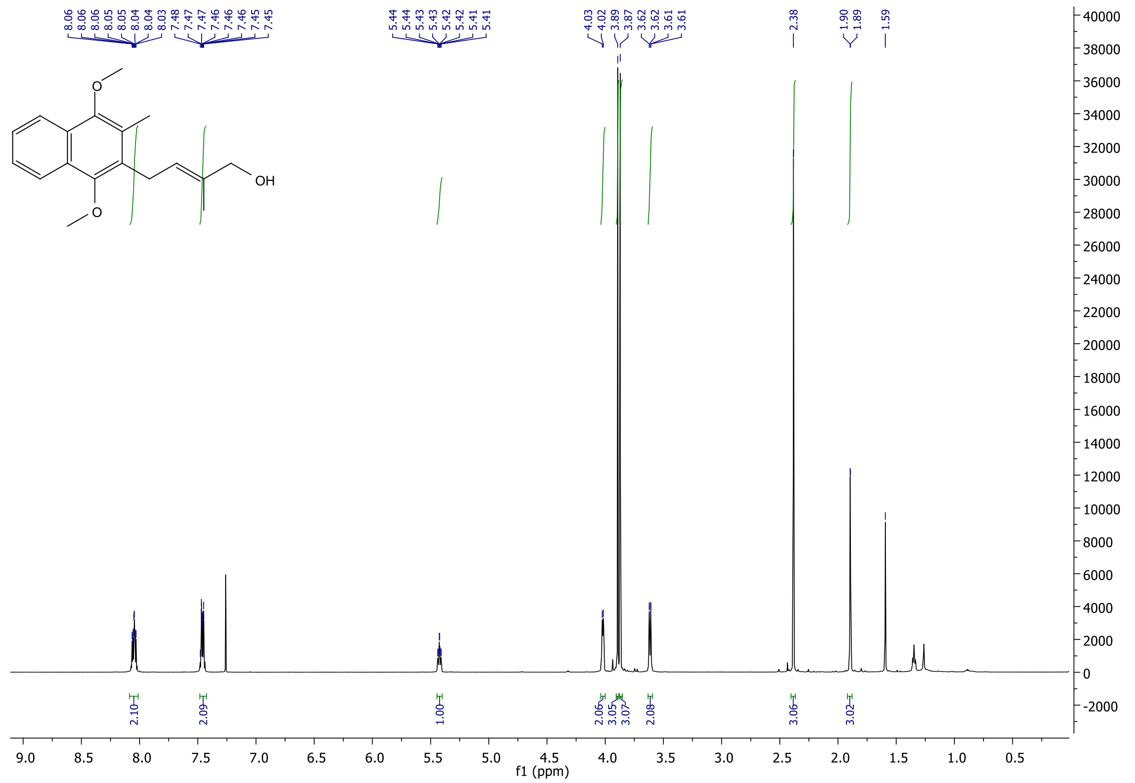

^{13}C NMR Spectrum (125 MHz, $CDCl_3$) of (*E*)-4-(1,4-dimethoxy-3-methylnaphthalen-2-yl)-2-methylbut-2-en-1-ol (**2e**)

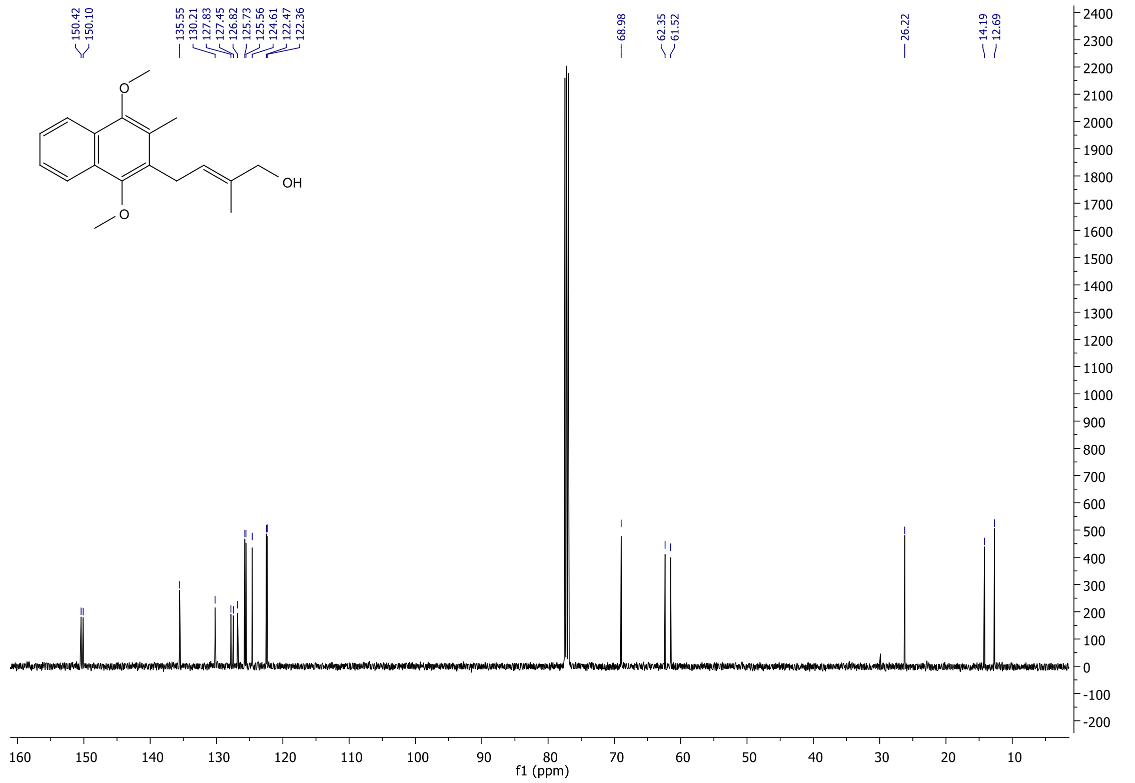

IR Spectrum of (*E*)-1,4-dimethoxy-2,3,5-trimethyl-6-(3-methyl-4-(phenylsulfonyl)but-2-enyl)benzene (**10**)

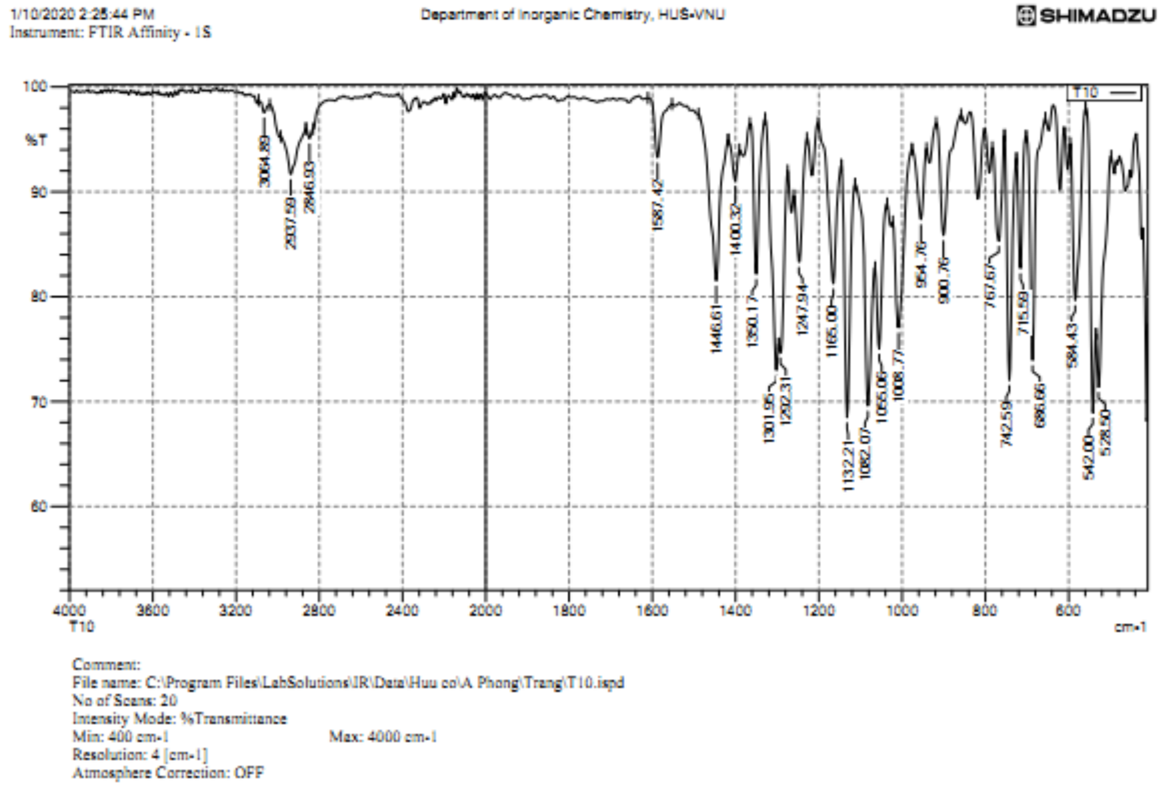

^{1}H NMR Spectrum (500 MHz, $CDCl_3$) of (*E*)-1,4-dimethoxy-2,3,5-trimethyl-6-(3-methyl-4-(phenylsulfonyl)but-2-enyl)benzene (**10**)

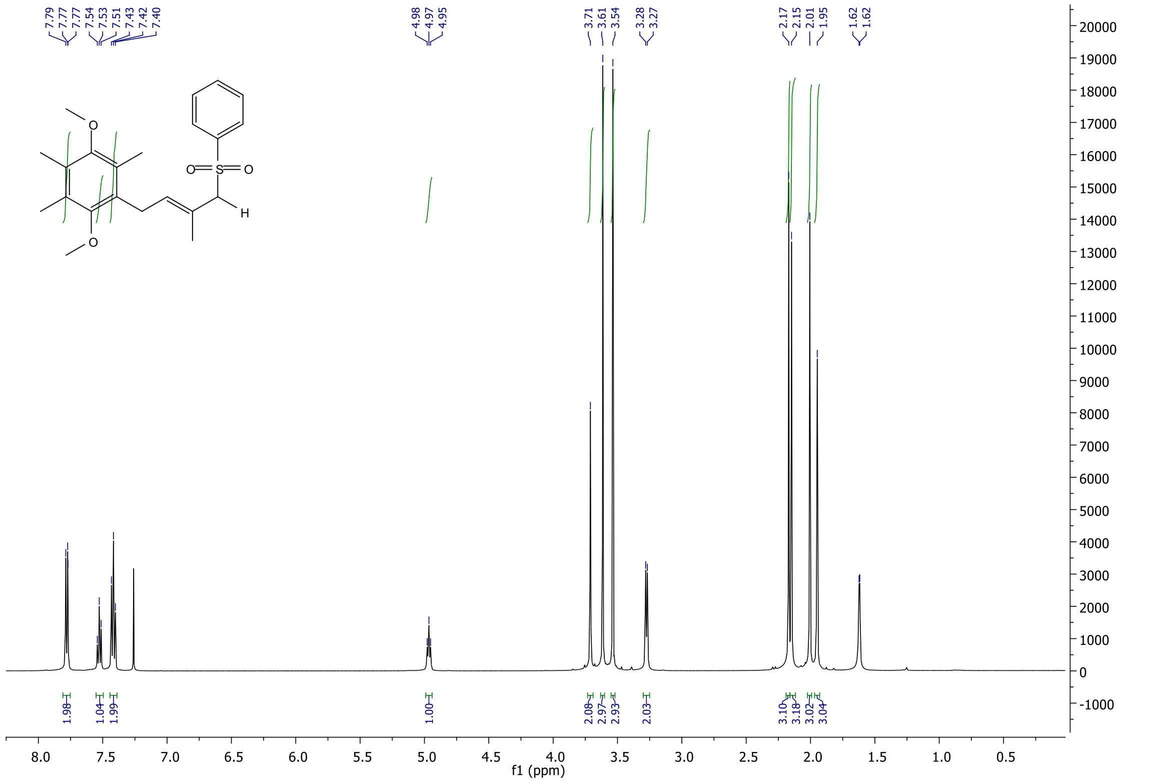

^{13}C NMR Spectrum (125 MHz, $CDCl_3$) of (*E*)-1,4-dimethoxy-2,3,5-trimethyl-6-(3-methyl-4-(phenylsulfonyl)but-2-enyl)benzene (**10**)

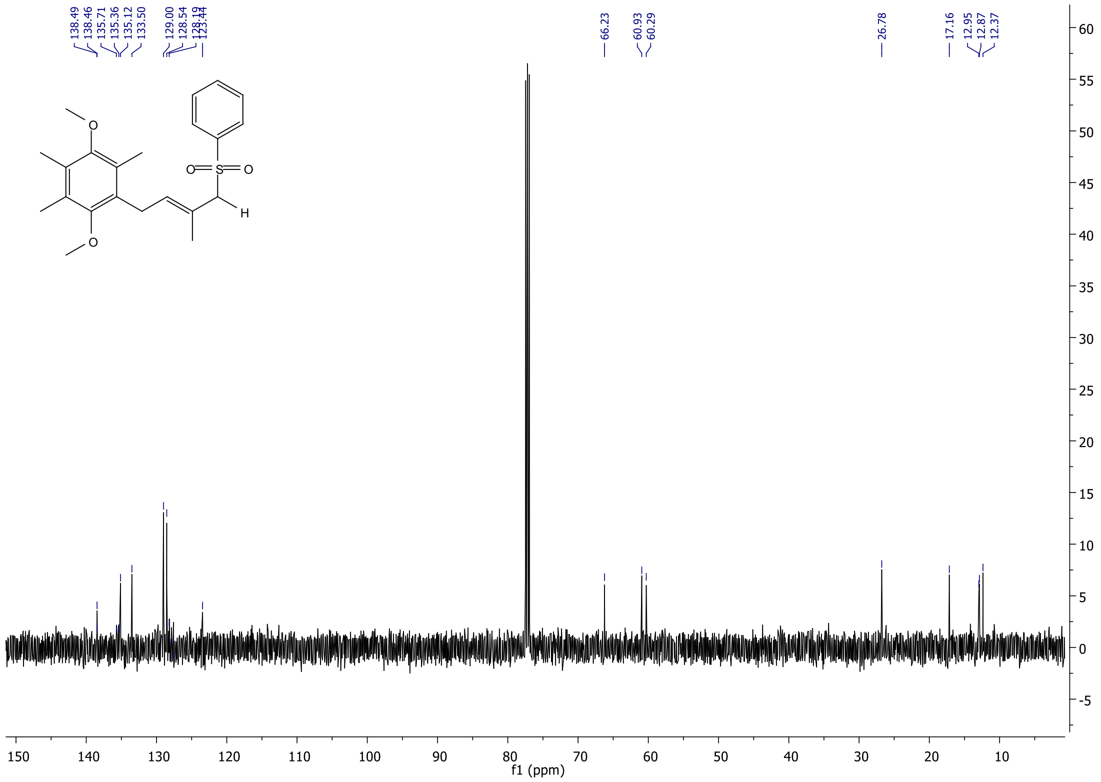

IR Spectrum of 1-((*2E,6E,10E,14E,18E,22E,26E,30E,34E*)-3,7,11,15,19,23,27,31,35,39-decamethyl-4-(phenylsulfonyl)tetraconta-2,6,10,14,18,22,26,30,34,38-decaenyl)-2,5-dimethoxy-3,4,6-trimethylbenzene (**11**)

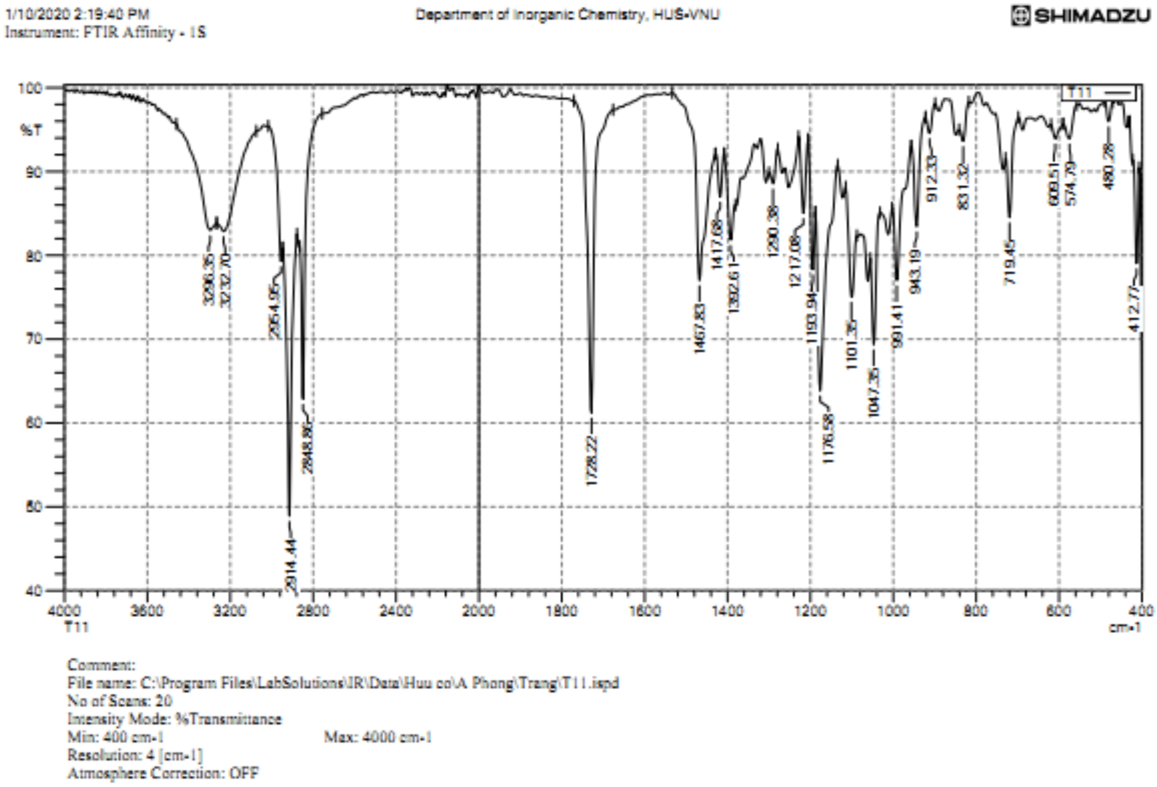

^{1}H NMR Spectrum (500 MHz, $CDCl_3$) of 1-((*2E,6E,10E,14E,18E,22E,26E,30E,34E*)-3,7,11,15,19,23,27,31,35,39-decamethyl-4-(phenylsulfonyl)tetraconta-2,6,10,14,18,22,26,30,34,38-decaenyl)-2,5-dimethoxy-3,4,6-trimethylbenzene (**11**)

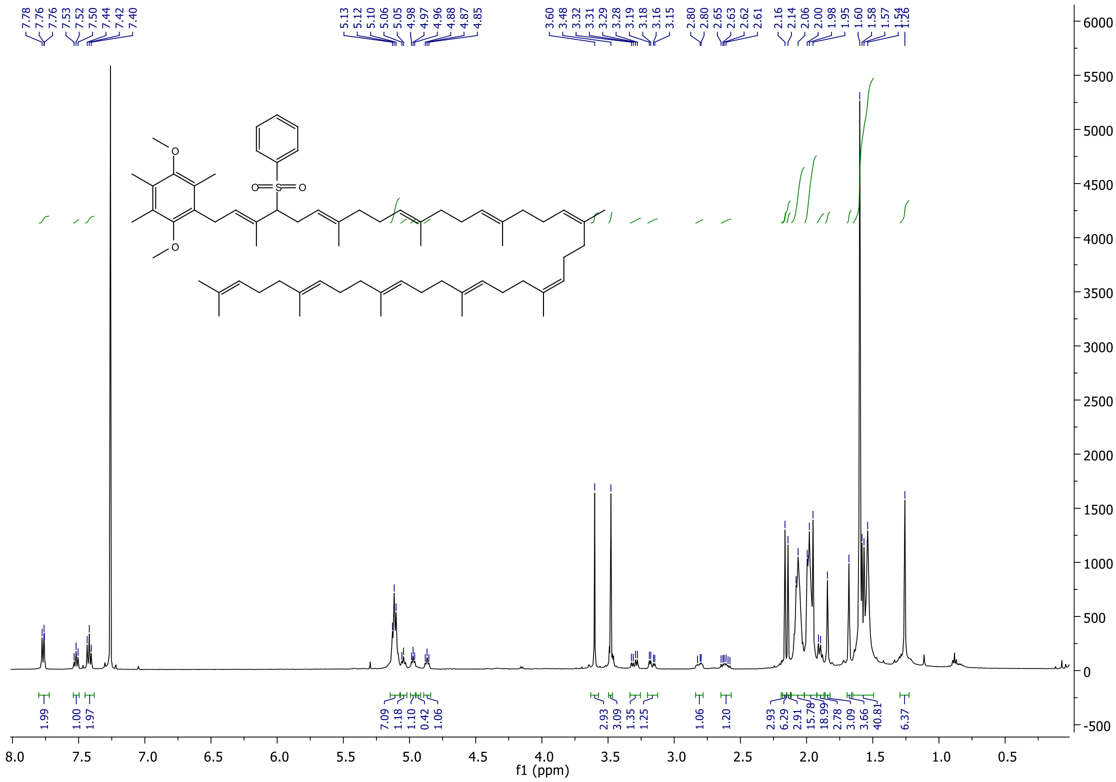

^{13}C NMR Spectrum (125 MHz, $CDCl_3$) of 1-((*2E,6E,10E,14E,18E,22E,26E,30E,34E*)-3,7,11,15,19,23,27,31,35,39-decamethyl-4-(phenylsulfonyl)tetraconta-2,6,10,14,18,22,26,30,34,38-decaenyl)-2,5-dimethoxy-3,4,6-trimethylbenzene (**11**)

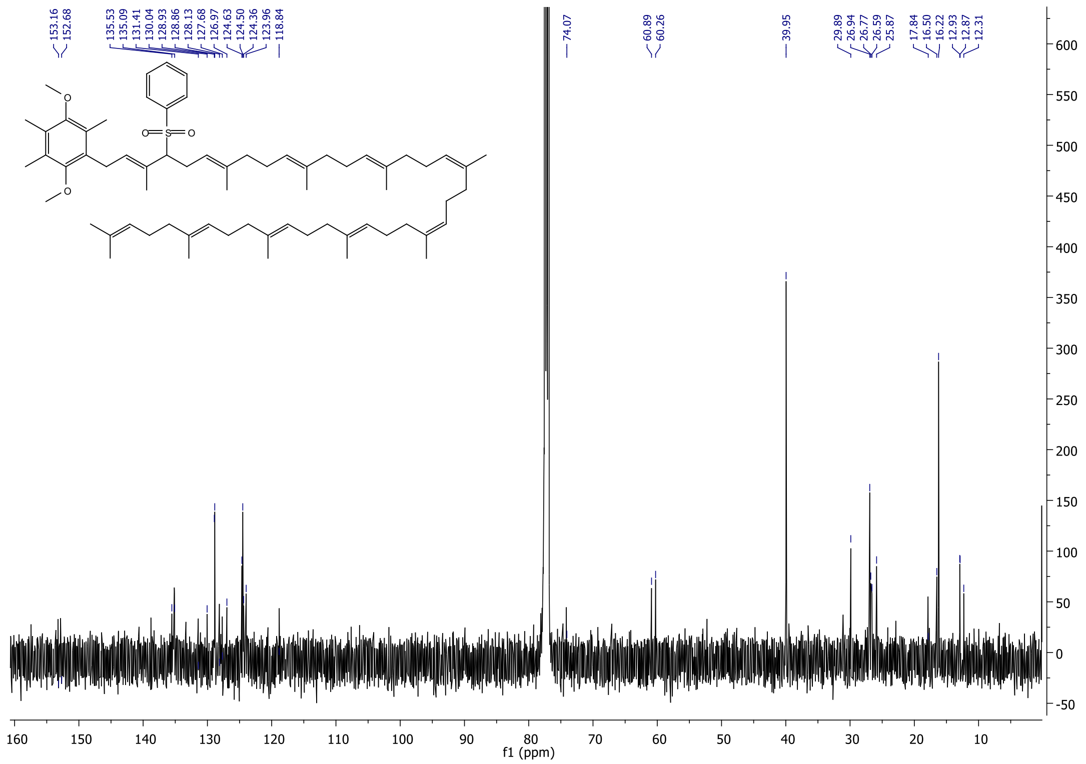

IR Spectrum of 1-((*2E,6E,10E,14E,18E,22E,26E,30E,34E*)-3,7,11,15,19,23,27,31,35,39-decamethyltetraconta-2,6,10,14,18,22,26,30,34,38-decaenyl)-2,5-dimethoxy-3,4,6-trimethylbenzene (**12**)

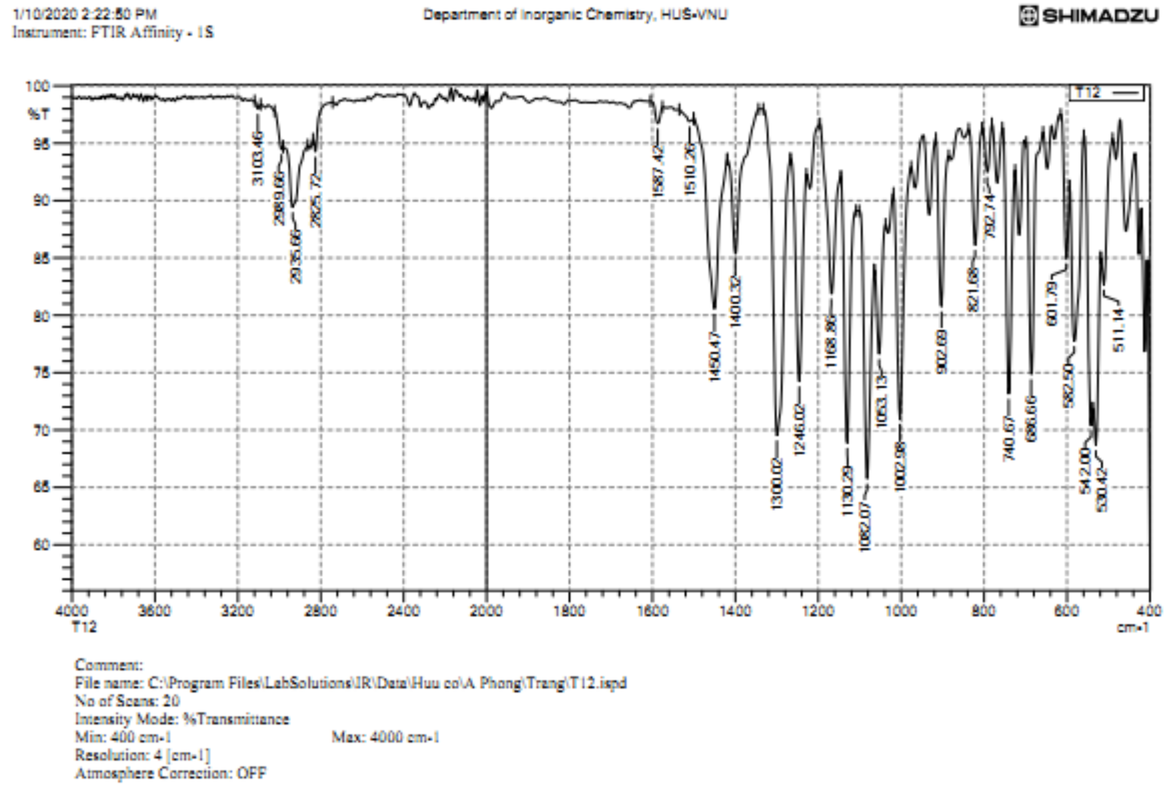

^{1}H NMR Spectrum (500 MHz, $CDCl_3$) of 1-((*2E,6E,10E,14E,18E,22E,26E,30E,34E*)-3,7,11,15,19,23,27,31,35,39-decamethyltetraconta-2,6,10,14,18,22,26,30,34,38-decaenyl)-2,5-dimethoxy-3,4,6-trimethylbenzene (**12**)

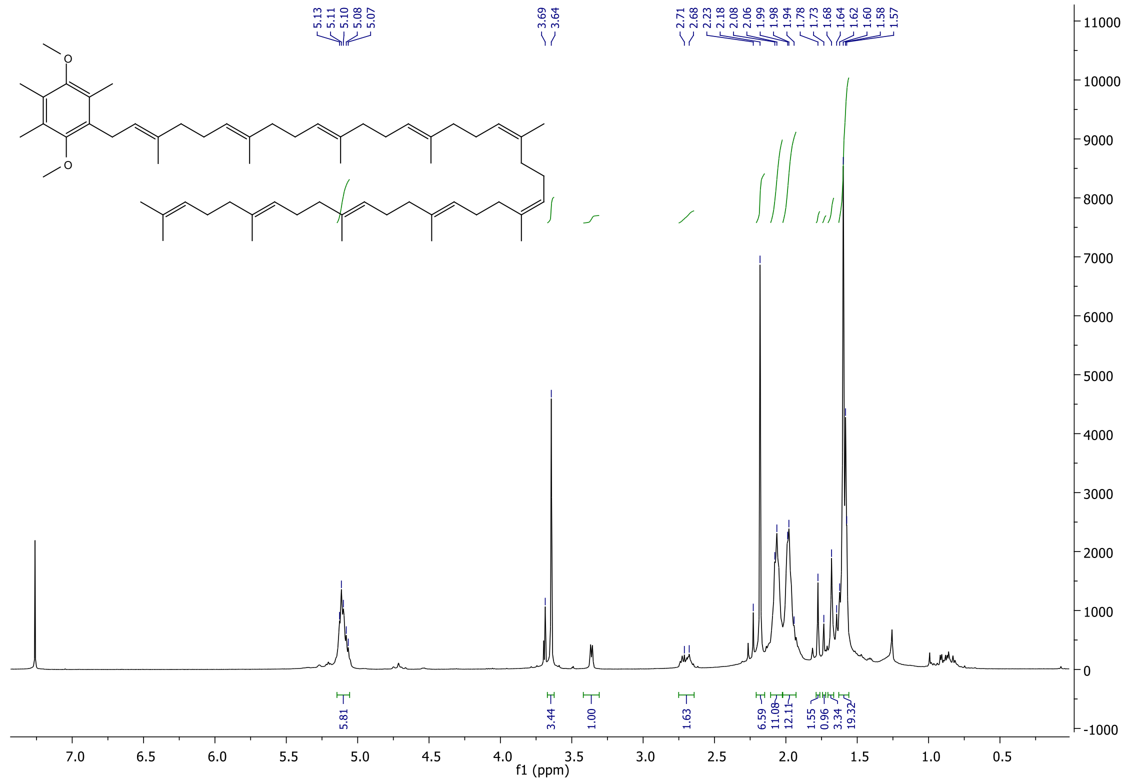

^{13}C NMR Spectrum (125 MHz, $CDCl_3$) of 1-((2E,6E,10E,14E,18E,22E,26E,30E,34E)-3,7,11,15,19,23,27,31,35,39-decamethyltetraconta-2,6,10,14,18,22,26,30,34,38-decaenyl)-2,5-dimethoxy-3,4,6-trimethylbenzene (**12**)

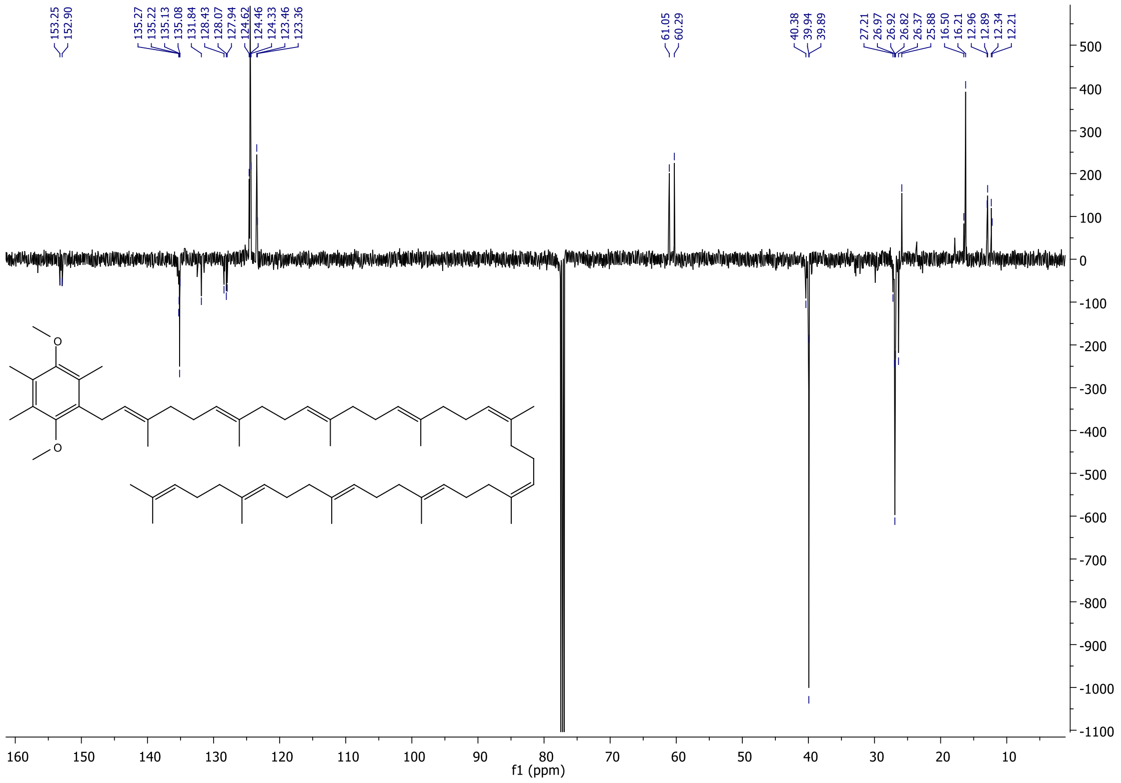

Supplement: Supplementary file 1 [file molecules-25-00448-s001.pdf]
